# Rumen-derived *Prevotella* and *Megasphaera elsdenii* mitigate methane production through functional modulation of rumen microbial metabolism

**DOI:** 10.1186/s40104-026-01460-5

**Published:** 2026-07-10

**Authors:** Banglin He, Beiyi Liu, Xin Wang, Min Xia, Chenglong Ding, Mudasir Nazar, Yanfen Cheng, Dingfu Xiao

**Affiliations:** 1 College of Animal Science and Technology, Hunan Agricultural University, Changsha, 410128 China; 2 Key Laboratory of Crop and Animal Integrated Farming, Ministry of Agriculture, Jiangsu Academy of Agricultural Science, Nanjing, 210014 China; 3 Institute of Animal Science, Jiangsu Academy of Agricultural Science, Nanjing, 210014 China; 4 Yuelushan Laboratory, Hunan Province Yuelushan Laboratory, Changsha, 410128, China; 5 College of Animal Technology, Nanjing Agricultural University, Nanjing, 210014, China

**Keywords:** *Megasphaera elsdenii*, Metagenomic sequencing, Methane emission, *Prevotella*, Rumen metabolism

## Abstract

**Background:**

Enteric methane (CH_4_) production represents a major energy loss in ruminant systems and contributes substantially to agricultural greenhouse gas emissions. Increasing ruminal propionate production has been proposed as a strategy to redirect metabolic hydrogen away from methanogenesis, although the underlying microbial mechanisms remain incompletely understood.

**Results:**

Four rumen-derived *Prevotella* strains and one *Megasphaera elsdenii* strain were isolated, genomically characterized, and evaluated using an in vitro rumen fermentation model. Distinct strain-specific responses were observed. Compared with the control, *Prevotella* strains RH14 and RH35 significantly reduced CH_4_ accumulation at 48 h (*P* < 0.05), coinciding with lower total gas and carbon dioxide (CO_2_) production, whereas RH3, RH27, and RH19 showed CH_4_ production comparable to the control. Volatile fatty acid (VFA) profiles showed comparatively smaller differences among treatments, although RH14 maintained relatively greater total VFA and propionate concentrations at later incubation stages. Metagenomic analysis indicated that methane mitigation was associated with reduced relative abundance of methanogenesis-related pathways, particularly hydrogenotrophic methanogenesis (*P* < 0.05), whereas archaeal community composition remained largely unchanged. However, metagenomic data reflect gene abundance rather than activity and do not directly indicate functional regulation.

**Conclusions:**

These findings demonstrate strain-specific effects of rumen-derived bacteria on rumen fermentation and methane production. In particular, *Prevotella* strains RH14 and RH35 showed potential to mitigate methane formation through functional modulation of microbial metabolism, partially displacing rather than completely eliminating methanogens. These results provide a functional basis for the future development of rumen microbial interventions aimed at improving rumen fermentation efficiency and mitigating enteric methane emissions.

**Graphical Abstract:**

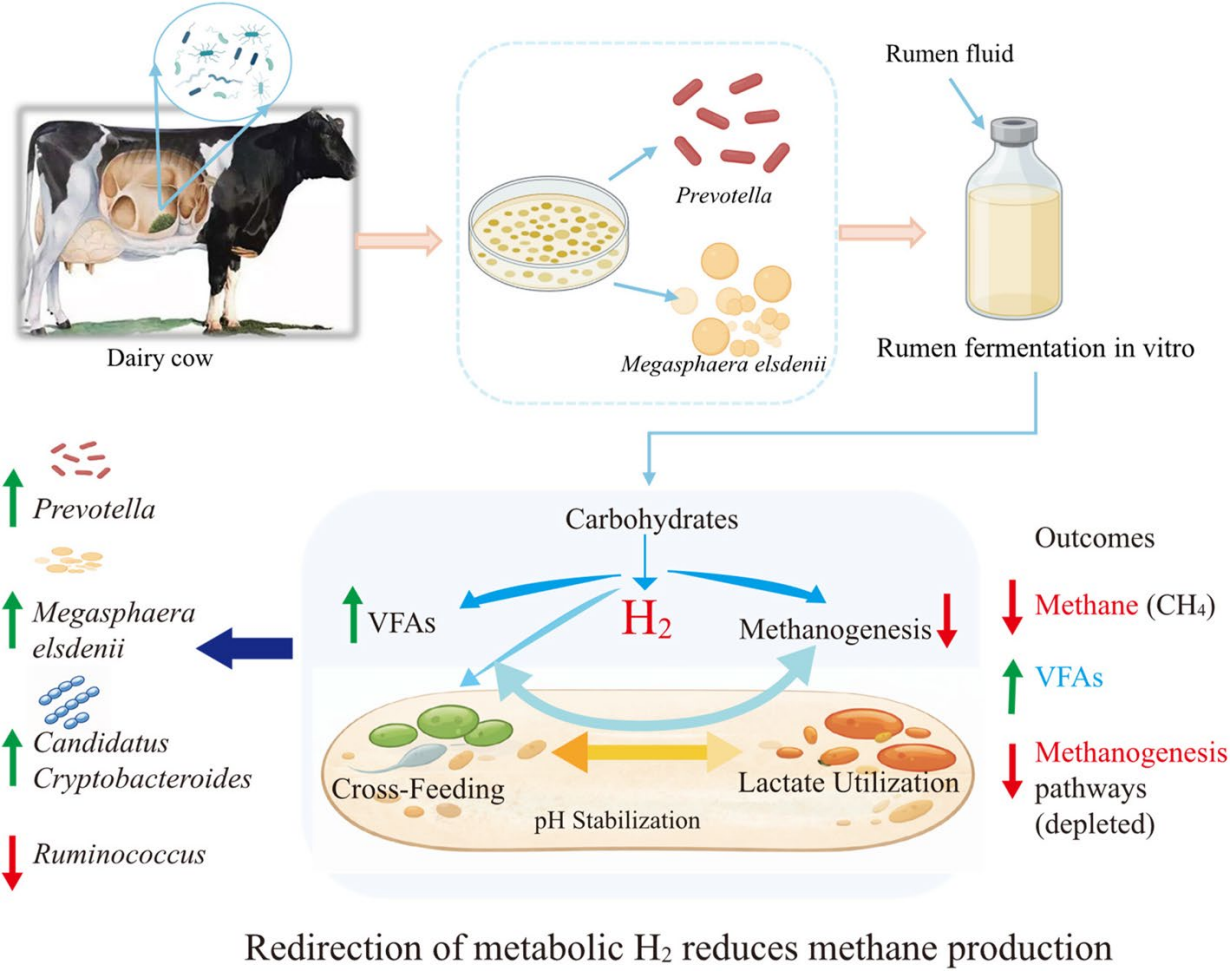

**Supplementary Information:**

The online version contains supplementary material available at 10.1186/s40104-026-01460-5.

## Introduction

As concerns over global warming continue to intensify, identifying effective strategies to mitigate greenhouse gas emissions has become a major focus of global research. The primary greenhouse gases include carbon dioxide (CO_2_), methane (CH_4_), and nitrous oxide (N_2_O), all of which contribute significantly to rising global surface temperatures [1]. Anthropogenic methane accounts for approximately 85% of global methane emissions, with agriculture contributing nearly 40% [2]. Among agricultural sources, ruminant livestock represents a major contributor, with enteric fermentation accounting for approximately 60% of methane emissions from this sector [3]. Although ruminants are important contributors to agricultural methane emissions, the dairy sector contributes approximately 2.7% of global anthropogenic greenhouse gas emissions [4]. Nevertheless, enteric methane production represents a substantial loss of dietary energy, which can negatively impact feed efficiency in dairy production systems.

The rumen fermentation system enables ruminants to efficiently utilize structural carbohydrates, as microorganisms degrade complex polysaccharides into volatile fatty acids (VFAs), which serve as the primary energy source for the host [5]. However, this process also produces CO_2_ and hydrogen (H_2_), which are subsequently utilized by methanogenic archaea, where CO_2_ is reduced to methane using H_2_ as the electron donor. Although this pathway contributes to maintaining rumen redox balance, methane production represents a loss of forage-derived energy and contributes to greenhouse gas emissions [6–8]. Therefore, strategies that redirect metabolic hydrogen ([H]) away from methanogenesis toward alternative sinks have received increasing attention.

Direct-fed microbials (DFM) have emerged as a promising approach to modulate rumen fermentation, as they are generally considered safe and are widely accepted in livestock production systems [9]. Importantly, the effectiveness of DFM is not solely dependent on introducing novel microorganisms, but also on enhancing the activity, abundance, or functional dominance of specific microbial groups that may already be present in the rumen but are limited by ecological or dietary constraints. In particular, the formulation of DFM using functionally complementary microbial strains represents a promising strategy to direct rumen fermentation toward more energetically favorable pathways. Such strategies are particularly relevant under conditions where the native microbial community is insufficient to efficiently utilize fermentation intermediates or redirect hydrogen flux, such as during dietary transitions or high-concentrate feeding.

*Prevotella* is a dominant genus in the rumen and plays an important role in carbohydrate degradation and propionate production. Although *Prevotella* is ubiquitously present, its abundance and metabolic activity can vary substantially depending on diet composition and physiological conditions [10–12]. Members of this genus primarily produce propionate via the succinate pathway, which consumes metabolic hydrogen and may contribute to decreased hydrogen availability for methanogenesis [13–15]. Several studies have also reported negative associations between *Prevotella* abundance and methane emissions, suggesting its potential role in modulating rumen fermentation toward reduced methane output [10, 16, 17].

In addition, *Megasphaera elsdenii*, a key lactate-utilizing bacterium in the rumen, contributes to propionate production via the acrylate pathway, particularly under high-concentrate feeding conditions [18]. This species is capable of metabolizing both L- and D-lactate isomers and may play an important role in stabilizing rumen fermentation and redirecting metabolic flux toward pathways that act as sinks for metabolic hydrogen, thereby indirectly limiting H_2_ formation during carbohydrate fermentation [19, 20].

Given the complementary metabolic pathways of *Prevotella* (succinate pathway) and *Megasphaera elsdenii* (acrylate pathway), supplementation with these bacteria may enhance propionate formation and promote hydrogen utilization through alternative pathways, thereby reducing methane production. Therefore, the present study aimed to evaluate, using an in vitro rumen fermentation system, whether supplementation with rumen-derived strains of *Prevotella* and *Megasphaera elsdenii* can effectively modulate fermentation patterns and reduce methane emissions. Furthermore, metagenomic analysis was conducted to assess the effects of these microbial interventions on the composition and functional potential of the rumen microbiome.

## Methods

### Animal feeding management

For single bacterial isolation and in vitro fermentation experiments, rumen fluid was collected from three lactating Holstein dairy cows fed a total mixed ration (TMR) with a forage-to-concentrate ratio of approximately 55:45 at Nanjing Weigang Dairy Co., Ltd. The detailed composition of the diets and the nutritional profile of the substrates used in the in vitro experiments are provided in Table S1. All procedures were approved by the Experimental Animal Welfare and Ethics Committee of Jiangsu Academy of Agricultural Sciences (IACUC-LE-2026-03-014).

### Rumen liquid collection and isolation of rumen bacteria

To avoid interference from feed, rumen contents were collected from three lactating dairy cows (611 ± 18.7 kg) using an oral tube after milking and before morning feeding. The contents were placed into a thermos flask, mixed, and transported back to the laboratory. After filtration through four layers of gauze, 1 mL of rumen fluid was aspirated and transferred into an anaerobic workstation (AW400SG, Electrotek, England). Gradient dilutions were performed using sterile physiological saline. Eight dilution gradients were prepared (10^−^^1^ to 10^−^^8^). From each dilution, 100 μL was aspirated and spread evenly onto kanamycin-vancomycin laked blood agar (KVLB, Haibo, China) solid medium. Each dilution gradient was plated in duplicate. Plates were incubated anaerobically at 37 °C for growth. After 24 h of incubation, distinct colonies were selected, re-diluted, and repeatedly streaked onto KVLB solid medium for purification. Following three rounds of purification, single bacterial strains were obtained. The bacterial strains ultimately isolated from the lactating cow rumen fluid were designated RH3, RH14, RH19, RH27, and RH35. The physiological and biochemical characteristics of the isolated strains were preliminarily determined using Gram staining and a Gram-negative bacteria identification system (Haibo, China). To assess the growth kinetics of the isolated strains, 1 mL of bacterial culture was inoculated into 150 mL of Schaedler broth (Solarbio, China). The flask was sealed and transferred from the anaerobic workstation to a shaking incubator. Bacterial culture samples were collected aseptically using a syringe at 2 h intervals. The optical density at 600 nm (OD_600_) of each sample was measured to plot the growth curve.

### Bacterial whole-genome analysis

#### Bacterial DNA extraction and 16S rRNA sequencing

Bacterial cells (1 mL) at the exponential growth phase were harvested, and genomic DNA was isolated using a commercial bacterial DNA extraction kit (Tiangen, Beijing, China). The extracted DNA served as the template for amplifying the 16S rRNA gene using PCR with the universal primers 27 F (5′-GAGTTTGATCTGGCTCAG-3′) and 1492R (5′-ACGGCTACCTTGTTACGACTT-3′). Each 20 μL PCR reaction contained 10 μL of PrimeSTAR Max Premix (2 ×, Takara Bio), 0.5 μL of each primer, 0.5 μL of genomic DNA, and 8.5 μL of nuclease-free water. PCR products were resolved on a 1% agarose gel, and amplicons showing a single band of the expected size were purified and submitted to Qingke Biotechnology (Nanjing, China) for Sanger sequencing. The obtained sequences were queried against the NCBI nucleotide database using BLAST. Multiple-sequence alignments and phylogenetic tree construction were performed in MEGA v12.0.0.

#### Library construction and NGS sequencing

Next-generation sequencing was carried out by Shanghai Biozeron Biotechnology Co., Ltd. (Shanghai, China). For paired-end NGS sequencing of each bacterial strain, at least 1 μg of high-quality genomic DNA was used for library preparation. Paired-end libraries with an approximate insert size of 400 bp were constructed following standard protocols for genomic DNA library generation. Briefly, purified genomic DNA was fragmented to the desired size using a Covaris system, and the resulting fragments were end-repaired with T4 DNA polymerase. An adenine residue was added to the 3′ termini of phosphorylated blunt-end fragments, after which sequencing adapters were ligated. Size-selected fragments were subsequently isolated by gel electrophoresis, followed by selective enrichment and amplification through PCR. Index sequences were introduced into the adapters during the PCR step when required, and library quality was assessed before sequencing. Libraries meeting quality criteria were subjected to paired-end sequencing (150 bp ×2) on the NGS platform at Shanghai Biozeron.

#### Genome assembly

Raw paired-end reads were subjected to quality trimming and filtering using Trimmomatic (version 0.36) [21] with parameters (SLIDINGWINDOW:4:15 MINLEN:75). The resulting high-quality reads were used for downstream analysis. De novo genome assembly was performed with ABySS 2.2.0 [22], employing multiple k-mer values to identify the optimal assembly. Finally, GapCloser [23] was utilized to close residual gaps and correct single-base polymorphisms within the draft assembly.

#### Genome annotation

For prokaryotic strains, gene models for *Prevotella* and *Megasphaera elsdenii* were predicted using an ab initio approach with GeneMark 4.17 [24] (http://topaz.gatech.edu/GeneMark/).

Predicted genes were then functionally annotated by performing BLASTp searches against multiple databases, including NCBI non-redundant (NR), SwissProt (http://uniprot.org), KEGG (http://www.genome.jp/kegg/), COG (http://www.ncbi.nlm.nih.gov/COG), CAZy (http://www.cazy.org/), CARD (https://card.mcmaster.ca/), PHI, TCDB (http://www.tcdb.org/), VFDB, BacMet, as well as SignalP (http://www.cbs.dtu.dk/services/SignalP/) and TMHMM (http://www.cbs.dtu.dk/services/TMHMM/) for signal peptide and transmembrane domain prediction. In addition, tRNA genes were identified using tRNAscan-SE v2.0.4 [25], and rRNA genes were detected with RNAmmer v1.2 [26].

### Batch culture of rumen fluid in vitro

Rumen fluid was collected from three lactating dairy cows as described above. Immediately after collection, equal volumes of rumen fluid from each cow were pooled to minimize individual animal variation and to obtain a representative inoculum. The pooled rumen fluid was filtered through four layers of cheesecloth under continuous CO_2_ flushing to maintain anaerobic conditions. For each fermentation, 20 mL of the filtered rumen fluid was mixed with 40 mL of artificial saliva prepared according to Menke et al. [27] in a 150-mL anaerobic fermentation bottle. The fermentation substrate consisted of 0.5 g of TMR obtained from the same dairy farm. Prior to incubation, the headspace of each bottle was flushed with CO_2_ to ensure anaerobic conditions, and the bottles were sealed with butyl rubber stoppers and aluminum caps. At the start of fermentation, 1 mL of bacterial suspension was added to each treatment bottle. The bacterial cultures were pre-grown to the exponential phase and adjusted to a concentration of 2 × 10^10^ CFU/mL [28]. The control group received 1 mL of sterile Schaedler broth instead. A pre-evacuated gas collection bag (E-Switch, Shanghai, China) was connected to each bottle prior to incubation. The bottles were incubated at 39 °C in a shaking incubator under anaerobic conditions. Gas production was measured at 12, 24, and 48 h, while fermentation samples were collected at 12, 24, 48, and 72 h. At each sampling time point, bottles were immediately placed on ice to terminate microbial activity, and pH was measured without delay.

Each treatment was conducted with three independent replicates (*n* = 3) using the same pooled rumen fluid inoculum.

### Determination of gas production volume, hydrogen and methane in samples

At the end of each fermentation period, the gas collection bag attached to the fermentation bottle was removed, and total gas production was quantified by measuring the displacement of the syringe plunger. A 0.5-mL aliquot of the collected gas was analyzed for hydrogen and methane using a gas chromatograph (Varian CP-3800, Varian Inc., Palo Alto, CA, USA). Separation was performed on a 13 × molecular sieve column (45–60 mesh; 2.0 mm × 3.2 mm × 2.0 mm, stainless steel) with a thermal conductivity detector. Instrument settings were as follows: oven temperature, 60 °C; injector and TCD temperature, 120 °C; flame ionization detector, 200 °C. Nitrogen was used as the carrier gas at a flow rate of 50 mL/min. Based on the measured gas composition, the contents of methane and carbon dioxide in the total gas were calculated.

### Determination of pH, NH_3_-N, MCP, and VFA

Immediately after the completion of fermentation, the pH of the fermentation broth was measured using a pH meter (FE28, Mettler-Toledo, Switzerland). Ammonia nitrogen (NH_3_-N) concentrations were determined via the phenol–hypochlorite colorimetric assay [29].

Microbial protein nitrogen (MCP) was determined using the purine method [30]. MCP (mg/mL) was calculated according to the formula: Microbial Protein Nitrogen (mg/mL) = (Measured RNA (mg/mL) × Nitrogen Content in RNA)/(Nitrogen Content in Bacterial RNA) × Dilution Factor. Subsequently, microbial protein concentration was calculated using the formula: Microbial Protein Concentration (mg/mL) = Microbial Protein Nitrogen (mg/mL) × 6.25.

The VFA concentration in the fermentation broth was determined according to the method described by Erwin et al. [31]. Briefly, 1 mL of 25% metaphosphoric acid was added to 5 mL of fermentation broth. After centrifugation (12,000 × *g*, 20 min, 4 °C), the supernatant was filtered through a 0.22-μm membrane. The filtrate was analyzed using a gas chromatograph (GC-14B, Shimadzu, Japan) equipped with a flame ionization detector (FID). Instrument settings were: column temperature 100 °C, detector temperature 200 °C, and injector temperature 200 °C.

Total VFA-carbon was calculated by weighting individual VFAs according to their carbon number (acetate ×2, propionate ×3, butyrate ×4, valerate ×5).

### DNA extraction, library construction, and metagenomic sequencing

Total genomic DNA was extracted from rumen fluid samples using the Mag-Bind^®^ Soil DNA Kit (Omega Bio-tek, Norcross, GA, USA) following the manufacturer’s instructions. DNA concentration and purity were assessed with TBS-380 and NanoDrop 2000, respectively, and integrity was verified by 1% agarose gel electrophoresis.

For library construction, genomic DNA was fragmented to an average size of ~400 bp using a Covaris M220 system (Gene Company Limited, China). Paired-end libraries were prepared using the NEXTFLEX Rapid DNA-Seq kit (Bioo Scientific, Austin, TX, USA), with adapters containing full sequencing primer sites ligated to blunt-ended fragments. Sequencing was performed on an Illumina NovaSeq platform (Illumina Inc., San Diego, CA, USA) at Majorbio Bio-Pharm Technology Co., Ltd. (Shanghai, China) using the NovaSeq 6000 S4 Reagent Kit v1.5 (300 cycles).

Raw paired-end reads were processed on the Majorbio Cloud Platform (www.majorbio.com). Adapter sequences and low-quality reads (length < 50 bp, quality score < 20, or containing N bases) were removed using fastp v0.20.0 [32]. Host-derived reads were filtered by aligning to the bovine reference genome with BWA v0.7.9a [33]. Remaining high-quality reads were assembled de novo using MEGAHIT v1.1.2 [34], and contigs ≥ 300 bp were retained for downstream analyses. Open reading frames (ORFs) were predicted from assembled contigs using Prodigal [35] or MetaGene [36]. With ORFs ≥ 100 bp translated into amino acid sequences based on the NCBI translation table. Redundant sequences were clustered with CD-HIT v4.6.1 [37] at 90% sequence identity and 90% coverage to generate a non-redundant gene catalog. Gene abundance was estimated by mapping reads to the catalog using SOAPaligner v2.21 [38] with 95% identity.

Representative sequences from the non-redundant catalog were taxonomically annotated using Diamond v0.8.35 [39] (http://www.diamondsearch.org/index.php) against the NCBI NR database (e-value ≤ 1 × 10^−5^). Concurrently, functional annotation was conducted by assigning Cluster of Orthologous Groups (COG) categories through alignment against the eggNOG database. The Kyoto Encyclopedia of Genes and Genome (KEGG) annotation was conducted using Diamond v0.8.35 [39] against the KEGG database (http://www.genome.jp/keeg/) with an e-value cutoff of 1 × 10^−5^. The CAZy annotation was performed using hmmscan (http://hmmer.janelia.org/search/hmmscan). The abundances of annotated KEGG Orthologs (KOs), pathways, enzymes, modules, and carbohydrate-active enzyme (CAZymes) were first quantified and normalized as counts per million reads (CPM). For subsequent analysis, we retained only those KEGG modules, pathways, enzymes, and CAZymes that exhibited a CPM value greater than 5 in at least 50% of the animals within any given experimental group.

### Statistical analysis

Gas composition and rumen fermentation parameters were analyzed using one-way ANOVA followed by Duncan's multiple range test in SPSS 22.0 (IBM, New York, USA). Differences in rumen microbial communities at the domain, phylum, genus, and species levels were assessed using the Kruskal–Wallis test for multiple comparisons, with *P* < 0.05 considered statistically significant. Similarly, the abundances of microbial metabolic pathways, modules, KEGG pathways and CAZymes across the six groups were compared using the Kruskal–Wallis test. Spearman rank correlation coefficients between rumen microbial taxa were calculated in SPSS 22.0, with *P* < 0.05 indicating significance.

## Results

### Isolation, identification, and genomic characterization of rumen bacteria

**Fig. 1:**
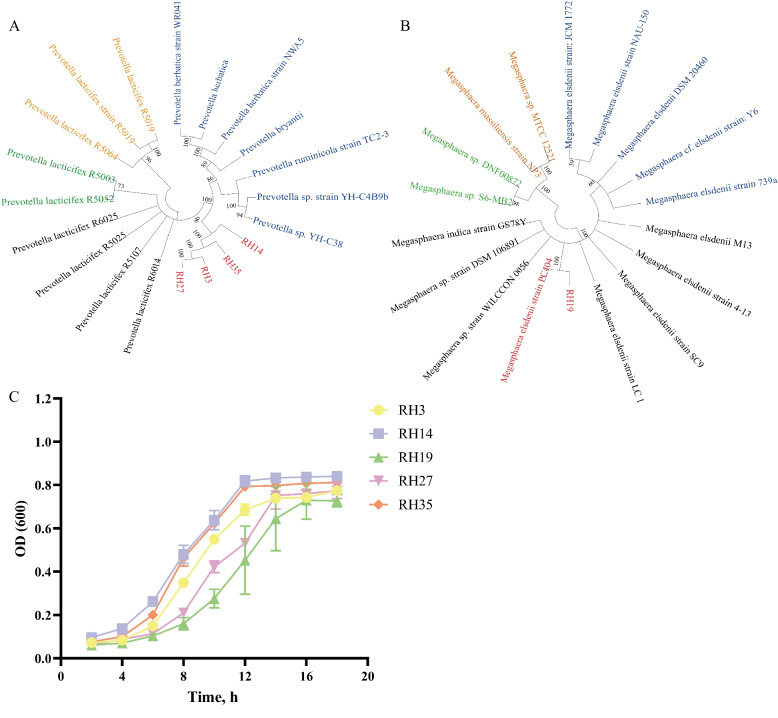
Phylogenetic identification and growth characteristics of four *Prevotella* strains and one *Megasphaera elsdenii* strain. **A** and **B **Phylogenetic analysis of four *Prevotella* strains and one *Megasphaera elsdenii* strain based on 16S rRNA gene sequences. The tree was constructed using the neighbor-joining method in MEGA 12, with bootstrap values calculated from 1,000 replicates and shown at the nodes. **C** Growth curves of *Prevotella* strains and *Megasphaera elsdenii* under anaerobic conditions. Growth was monitored by measuring optical density at 600 nm (OD₆₀₀) at the indicated time points. Values are expressed as mean ± SEM

A total of five bacterial strains were isolated from rumen fluid, including four *Prevotella* strains (RH3, RH14, RH27, RH35) and one *Megasphaera elsdenii* strain (RH19). Phylogenetic analysis of 16S rRNA sequences confirmed the taxonomic affiliations (Fig. 1A and B). Morphological and Gram staining characteristics were consistent with these identifications: *Prevotella* strains formed short-rod, Gram-negative colonies, whereas RH19 appeared as Gram-negative cocci (Fig. S1A and B). Growth curves revealed that *Prevotella* strains reached peak density earlier (12–14 h) than RH19 (16 h) under anaerobic conditions (Fig. 1C). Biochemical assays indicated substrate-specific fermentation capabilities, with *Prevotella* strains metabolizing various carbohydrates and *M. elsdenii* RH19 utilizing select amino acids and sugars (Table S2).

Whole-genome sequencing revealed genome sizes ranging from 2.43 Mb (RH19) to 5.24 Mb (RH3) with GC contents of 37%–53% (Table S3; Fig. S1C). CAZyme analysis indicated that *Prevotella* strains possessed more carbohydrate esterases, while RH19 had a higher abundance of glycosyltransferases but lacked polysaccharide lyases (Fig. S2). KEGG and eggNOG annotations further highlighted that *Prevotella* strains were enriched in carbohydrate metabolism pathways, whereas RH19 had more genes involved in amino acid metabolism (Fig. S3A and B). These genomic features suggest complementary metabolic capacities between *Prevotella* and *M. elsdenii*, which may indicate the potential for synergistic interactions during rumen fermentation.

### In vitro rumen fermentation characteristics

Clear strain-specific differences were observed in fermentation gas kinetics following bacterial supplementation (Table 1). At 12 h, distinct differences among strains were evident; however, this pattern did not persist uniformly at later time points. At 24 h and 48 h, RH14 and RH35 did not show higher total gas and CO_2_ accumulation compared with NC, but instead exhibited substantially lower gas production (*P* < 0.01). In contrast, RH3, RH27, and RH19 generally maintained higher total gas and CO_2_ production than NC at multiple time points.

**Table 1:** Effects of supplemented rumen-derived bacteria on in vitro rumen fermentation parameters

Items^1^	Sampling time, h	Treatments^2^						SEM^3^	*P*-values
		NC	RH3	RH14	RH27	RH35	RH19		
Gas production, mL	12	16.33^c^	41.70^a^	30.63^b^	41.17^a^	31.17^b^	29.17^b^	2.14	< 0.01
	24	33.57^b^	43.37^a^	31.43^b^	40.47^a^	30.27^b^	39.17^a^	1.32	< 0.01
	48	32.50^b^	43.40^a^	31.80^b^	42.00^a^	30.40^b^	42.67^a^	1.49	< 0.01
CH_4_, mL	12	0.99^d^	2.12^a^	1.46^c^	1.65b^c^	1.77^b^	1.52^bc^	0.09	< 0.01
	24	2.44^ab^	2.40^ab^	1.60^c^	2.48^a^	2.07^b^	2.22^ab^	0.08	0.01
	48	4.45^a^	4.07^a^	2.08^b^	4.22^a^	2.65^b^	4.08^a^	0.28	< 0.01
CO_2_, mL	12	15.33^c^	39.56^a^	29.17^b^	39.52^a^	29.39^b^	27.65^b^	2.06	< 0.01
	24	30.28^b^	39.76^a^	29.03^b^	36.55^a^	27.16^b^	35.83^a^	1.23	< 0.01
	48	28.04^b^	39.32^a^	29.71^b^	37.16^a^	27.75^b^	37.87^a^	1.30	< 0.01
pH	12	6.64^a^	6.57^b^	6.56^b^	6.57^b^	6.55^b^	6.56^b^	0.01	< 0.01
	24	6.63^a^	6.51^c^	6.57^b^	6.57^b^	6.62^ab^	6.57^b^	0.01	0.01
	48	6.62^a^	6.50^c^	6.55^b^	6.66^a^	6.64^a^	6.66^a^	0.02	< 0.01
	72	6.62^a^	6.51^b^	6.63^a^	6.62^a^	6.65^a^	6.63^a^	0.01	< 0.01
NH_3_-N, mg/dL	12	12.76^ab^	13.79^a^	12.25^b^	9.03^c^	11.95^b^	12.89^ab^	0.42	< 0.01
	24	18.99^c^	23.19^a^	21.90^ab^	18.86^c^	18.12^c^	20.57^bc^	0.38	0.01
	48	28.80	30.12	32.33	29.92	31.25	30.80	0.58	0.67
	72	28.76^d^	33.59^a^	33.37^ab^	29.77^ cd^	32.25^abc^	30.75^bcd^	0.52	< 0.01
MCP, mg/mL	12	3.44^bc^	4.27^a^	2.75^d^	3.29^c^	3.91^ab^	3.79^b^	0.13	< 0.01
	24	3.82^ab^	3.52^b^	3.58^b^	3.80^ab^	4.02^ab^	4.32^a^	0.07	0.02
	48	1.87^ab^	1.93^a^	1.93^a^	1.72^c^	1.44^d^	1.75^bc^	0.04	< 0.01
	72	1.64^c^	1.80^bc^	2.17^a^	1.92^abc^	2.05^ab^	1.87^abc^	0.05	0.04

^a–d ^Means bearing different superscripts in the same row differ significantly (*P* < 0.05)

^1^*CH*_4_ Methane, *CO*_2_ Carbon Dioxide, *MCP* Microbial crude protein

^2^*NC* Negative control, *RH3 Prevotella* RH3, *RH14 Prevotella* RH14, *RH27 Prevotella* RH27, *RH35 Prevotella* RH35, *RH19 Megasphaera elsdenii* RH19. Data were analyzed using the one-way ANOVA procedure (*n* = 3)

^3^*SEM* Standard error of the mean

One of the most prominent findings was the contrasting response of CH_4_ production among strains. RH14 and RH35 consistently reduced CH_4_ accumulation relative to NC, RH3, RH27, and RH19, and the differences became more pronounced as incubation progressed (*P* < 0.05). At 48 h, RH14 and RH35 reduced CH_4_ production by 53.3% and 40.4%, respectively, compared with NC. In contrast, RH3, RH27, and RH19 produced CH_4_ levels comparable to the control treatment. Reduced CH_4_ production in RH14 and RH35 coincided with lower total gas and CO_2_ accumulation. In contrast, RH3 and RH27 also showed greater gas, CO_2_, and TVFA production during several incubation stages. RH19 showed a similar fermentation pattern to RH3 and RH27 despite belonging to a different bacterial genus. These responses differed among supplemented strains. Fermentation pH differed among treatments throughout incubation and showed a gradual decline over time in all groups (*P* < 0.05). RH3 consistently exhibited the lowest pH values, whereas RH27, RH35, and RH19 maintained pH values similar to or slightly greater than NC during the later incubation stages. NH_3_-N concentrations also differed among treatments at 12, 24, and 72 h (*P* < 0.05), but not at 48 h. RH3 showed greater NH_3_-N concentrations at 24 and 72 h compared with several other treatments.

MCP concentrations differed among treatments at all incubation stages (*P* < 0.05). RH3 and RH19 stimulated relatively greater MCP production during the early fermentation period, whereas MCP concentrations declined in all treatments at 48 h. At 72 h, RH14 and RH35 maintained numerically greater MCP concentrations than NC (*P* < 0.05).

Volatile fatty acid concentrations were strongly influenced by incubation time and differed among bacterial strains (Table 2; Fig. S4). TVFA concentrations differed among treatments at 12, 24, and 72 h (*P* < 0.05), but not at 48 h (*P* > 0.05). Total VFA concentrations increased from 12 to 48 h in all treatments and subsequently declined at 72 h, with a greater decrease observed in NC. However, because CH_4_ production was not measured at 72 h, the relationship between the decline in VFA concentrations and methane accumulation during the later fermentation stage could not be evaluated.

**Table 2:** Effects of supplemented rumen-derived bacteria on VFAs in rumen fermentation broth in vitro

Items^1^	Sampling time, h	Treatments^2^						SEM^3^	*P*-values
		NC	RH3	RH14	RH27	RH35	RH19		
TVFA, mmol/L	12	76.05^b^	93.24^a^	79.47^b^	64.37^b^	71.39^b^	77.78^b^	2.87	0.01
	24	79.65^b^	96.41^a^	79.69^b^	78.73^b^	92.49^a^	97.33^a^	2.15	< 0.01
	48	98.69	101.39	104.80	98.54	103.50	102.61	1.19	0.63
	72	82.35^d^	104.69^a^	101.67^a^	89.05^c^	94.68^bc^	95.43^b^	1.92	< 0.01
Acetate, mmol/L	12	43.30	51.69	46.26	37.64	41.63	45.40	1.47	0.09
	24	45.11	50.29	46.11	44.66	49.97	55.85	1.39	0.14
	48	61.57	62.09	66.10	57.20	59.83	61.39	1.25	0.52
	72	50.09^d^	64.07^a^	60.43^ab^	54.64^ cd^	56.58^bc^	56.58^bc^	1.22	< 0.01
Propionate, mmol/L	12	15.78^b^	24.26^a^	26.35^b^	13.18^b^	14.28^b^	15.86^b^	1.00	< 0.01
	24	15.66^c^	19.68^ab^	16.89^bc^	16.52^c^	17.35^abc^	19.95^a^	0.50	0.03
	48	17.63^c^	21.19^a^	22.86^a^	18.21^bc^	18.39^bc^	19.09^ab^	0.64	0.01
	72	16.22^d^	19.66^ab^	20.35^a^	17.05^c^	18.46^abc^	18.01^bc^	0.41	< 0.01
Butyrate, mmol/L	12	9.49	11.13	9.13	7.40	7.92	9.17	0.39	0.07
	24	10.09^abc^	12.85^a^	9.65^bc^	9.07^c^	10.49^abc^	12.25^ab^	0.44	0.04
	48	10.65	10.87	11.91	12.28	11.47	12.38	0.31	0.54
	72	9.10^b^	11.90^a^	12.07^a^	10.65^ab^	12.10^a^	12.18^a^	0.36	0.04
Valerate, mmol/L	12	1.84	2.32	1.45	1.48	1.52	2.13	0.13	0.29
	24	1.70	2.33	1.59	1.95	1.38	2.67	0.16	0.18
	48	2.35	1.79	2.29	2.72	1.86	2.41	0.16	0.58
	72	1.59	2.09	2.01	1.82	2.34	2.09	0.11	0.59
Acetate/propionate	12	2.76^ab^	2.13^b^	2.95^a^	2.87^ab^	2.92^a^	2.88^ab^	0.10	< 0.01
	24	2.90	2.55	2.75	2.69	2.88	2.82	0.07	0.74
	48	3.49	2.97	2.90	3.15	3.25	3.25	0.08	0.25
	72	3.09	3.28	2.98	3.21	3.06	3.14	0.05	0.70

^a–d ^Means bearing different superscripts in the same row differ significantly (*P* < 0.05)

^1^*TVFA* Total volatile fatty acids

^2^*NC* Negative control, *RH3 Prevotella* RH3, *RH14 Prevotella* RH14, *RH27 Prevotella* RH27, *RH35 Prevotella* RH35, *RH19 Megasphaera elsdenii* RH19. Data were analyzed using the one-way ANOVA procedure (*n* = 3)

^3^*SEM* Standard error of the mean

Compared with the clear differences observed for CH_4_, total gas, and CO_2_ production, VFA profiles showed comparatively smaller differences among treatments. RH3, RH35, and RH19 generally showed greater TVFA concentrations than NC during several incubation stages, whereas RH14 maintained relatively high TVFA concentrations at 48 and 72 h. Similar trends were also observed for TVFA-carbon concentrations, with significant differences detected among treatments during several incubation stages (*P* < 0.05) (Fig. S4). Acetate concentrations differed among treatments only at 72 h (*P* < 0.01), whereas propionate concentrations differed throughout incubation (*P* < 0.05). However, acetate and propionate proportions remained relatively stable among treatments during most incubation stages.

Some strain-specific differences were also observed for individual VFAs. RH3 showed greater propionate concentrations than NC during the early incubation period, whereas RH14 maintained relatively high propionate concentrations at 48 and 72 h. Butyrate concentrations differed among treatments at 24 and 72 h (*P* < 0.05), whereas valerate concentrations and acetate-to-propionate ratios showed comparatively smaller treatment effects during most incubation stages (*P* > 0.05).

### Metagenomic analysis of in vitro supplementation with *Prevotella* and *Megasphaera elsdenii*

Metagenomic sequencing generated a total of 1,302,559,334 reads, with 72,364,407 ± 930,848 reads per sample (mean ± SEM). Furthermore, a total of 1,294,690,664 reads were retained, with 71,927,259 ± 929,658 reads per sample (Table S4).

**Fig. 2:**
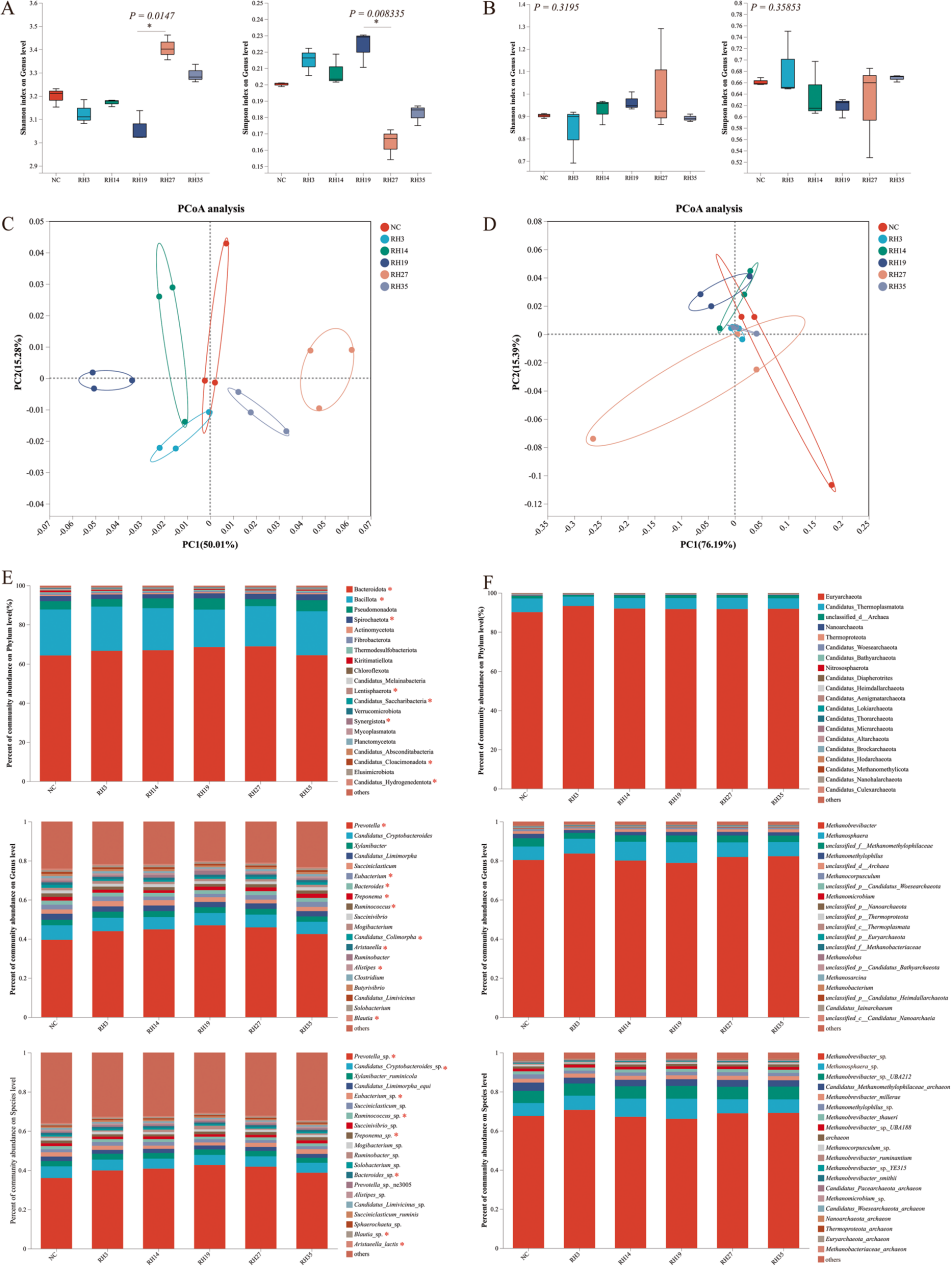
Effects of *Prevotella* and *Megasphaera elsdenii* on rumen microbial community composition and diversity in the in vitro fermentation model. **A** Bacteria alpha diversity. **B** Archaea alpha diversity. **C** Bacteria beta diversity (PCoA based on Bray–Curtis distance). **D** Archaea beta diversity (PCoA based on Bray–Curtis distance). **E** Relative abundance of bacteria communities at phylum, genus, and species levels. **F** Relative abundance of archaea communities at phylum, genus, and species levels. The difference among six groups was identified by Kruskal–Wallis multiple comparisons, and asterisk indicated the significant difference (*P* < 0.05)

Comparative analysis of the rumen microbiota across six treatments revealed no significant differences at the microbial domain level. Bacteria, Eukaryota, and Viruses showed no statistically significant variations among treatments (*P* > 0.05; Table 3). In addition, the relative abundance of Archaea was 15%–26% lower in the treatments compared to the control. However, these differences were not statistically significant (*P* > 0.05). For bacterial α-diversity, the Shannon index was significantly higher in the RH27 treatment compared to RH19 (*P* < 0.05), while the Simpson index was significantly higher in RH19 than in RH27 (*P* < 0.05). Additionally, no significant differences were observed in other α-diversity indices among the remaining treatments (Fig. 2A). Regarding archaea α-diversity (Fig. 2B), no significant differences were observed across any treatments (*P* > 0.05). Furthermore, PCoA analysis of bacterial β-diversity revealed significant separation between in vitro supplementation groups of *Prevotella* and *Megasphaera elsdenii* based on Bray–Curtis distances. The first principal coordinate (PC1) accounted for 50.01% of variation, while PC2 explained 15.28% (Fig. 2C). For archaeal communities, PCoA analysis showed that PC1 and PC2 explained 76.19% and 15.39% of variation, respectively (Fig. 2D).

**Table 3:** Relative abundance of microbial domains among NC, RH3, RH14, RH19, RH27 and RH35 rumen fermentation samples

Items, %	Dietary treatment^a^						SEM^b^	*P*-value
	NC	RH3	RH14	RH27	RH35	RH19		
Bacteria	94.59	95.24	95.23	95.68	95.09	95.40	0.129	0.238
Archaea	3.40	2.83	2.90	2.52	2.86	2.71	0.112	0.504
Eukaryota	0.032	0.045	0.047	0.043	0.051	0.051	0.002	0.329
Viruses	1.98	1.89	1.83	1.75	2.00	1.84	0.045	0.127

^a^Relative abundances of microbial domains (Bacteria, Archaea, Eukaryota, and Viruses) are expressed as percentages of total annotated sequences. *NC* negative control, RH3, RH14, RH27, and RH35 = *Prevotella* strains, *RH19 Megasphaera elsdenii*. Data are presented as means of three biological replicates (*n* = 3). Data were analyzed using the Kruskal–Wallis multiple comparisons, and significance was declared at *P* < 0.05

^b^*SEM* Standard error of the mean

### Abundance characteristics and taxonomic differences of rumen microbiota

**Fig. 3:**
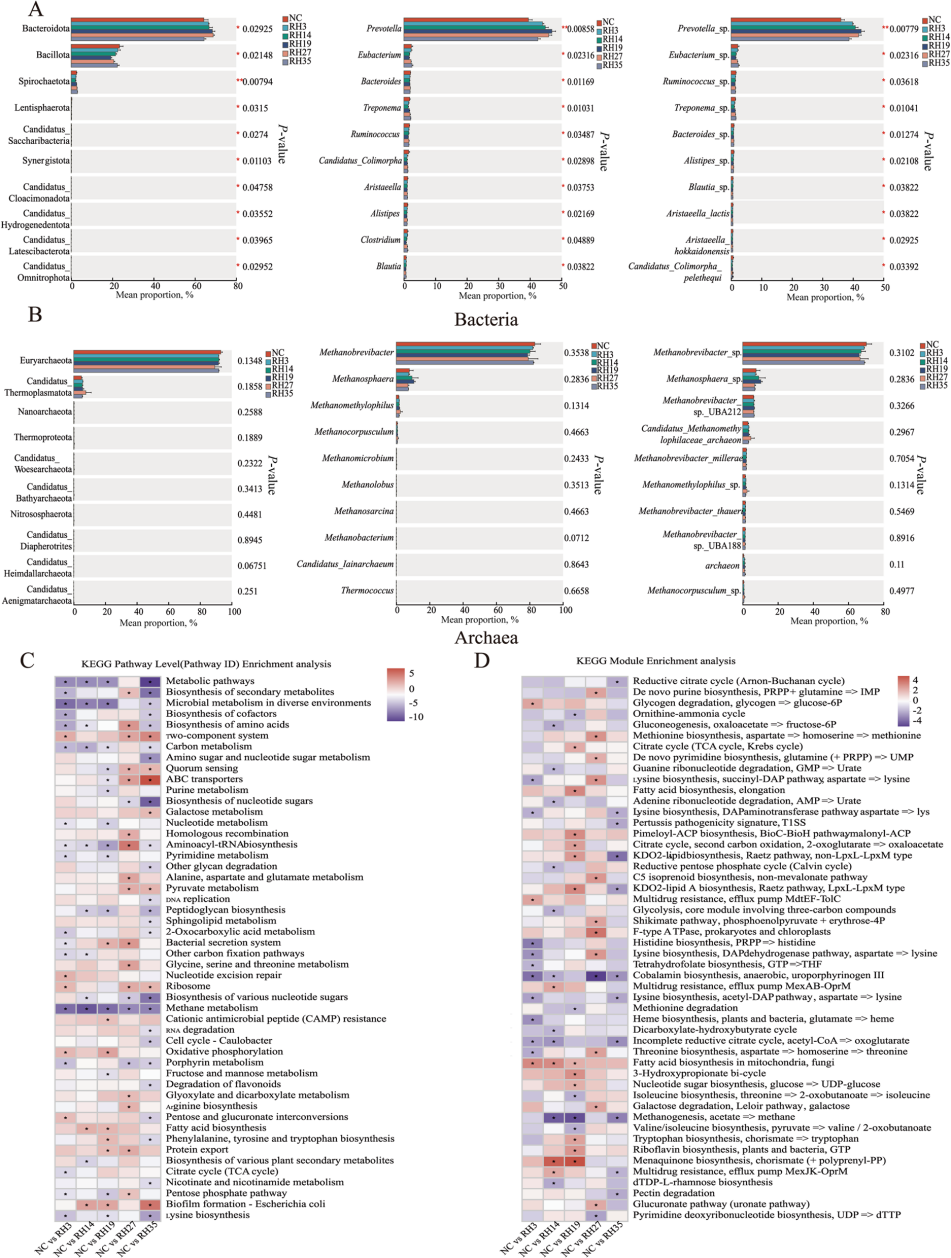
Differential analysis of rumen microbial communities and KEGG functional profiles following in vitro supplementation with *Prevotella* and *Megasphaera elsdenii*. **A** Differential abundance analysis of bacteria at phylum, genus, and species levels. **B** Differential abundance analysis of archaea at phylum, genus, and species levels. Asterisks indicate significant differences (*P* < 0.05). **C** Comparison of rumen microbial KEGG metabolic pathways across different treatment groups. **D** Comparison of rumen microbial KEGG modules across different treatment groups. Purple indicates enrichment in the control group (NC); red indicates enrichment in treatment groups. Asterisks denote pathways with reporter scores > 1.65 or < −1.65. The Kruskal–Wallis multiple comparisons were used for mean comparison

For bacterial composition (Fig. 2E), predominant phyla included Bacteroidetes (66.58% ± 1.14%), Bacillota (21.64% ± 0.90%), and Pseudomonadota (4.66% ± 0.66%). Dominant genera comprised *Prevotella* (43.95% ± 0.92%), *Candidatus*_*Cryptobacteroides* (6.62% ± 0.46%), *Xylanibacter* (2.93% ± 0.14%), *Candidatus*_*Limimorpha* (2.81% ± 0.12%), and *Succiniclasticum* (2.36% ± 0.31%). Prevalent species featured *Prevotella*_sp. (39.97% ± 0.83%), *Candidatus*_*Cryptobacteroides*_sp. (5.36% ± 0.41%), *Xylanibacter*_*ruminicola* (2.77% ± 0.13%), *Candidatus_Limimorpha_equi* (2.06% ± 0.10%), and *Eubacterium*_sp. (2.01% ± 0.20%). In the differential abundance analysis of rumen microbiota (Fig. 3A) at the phylum level, RH3 and RH19 significantly increased Bacteroidetes abundance compared to the NC treatment (*P* < 0.05), while significantly decreasing Bacillota abundance (*P* < 0.05). Additionally, RH14 treatment also exhibited significantly lower Bacillota abundance than NC (*P* < 0.05). Furthermore, RH14 showed significantly reduced *Spirochaetota* abundance relative to all other treatments (*P* < 0.05). At the genus level, *Prevotella* abundance was significantly higher in RH3, RH14, and RH19 treatments compared to NC group (*P* < 0.05). Additionally, *Eubacterium* abundance in RH14 and RH19 was significantly lower than in all other treatments (*P* < 0.05). For *Treponema* abundance, all treatments except RH14 showed significantly higher levels than NC (*P* < 0.05), while RH14 exhibited significantly reduced abundance (*P* < 0.05). Regarding *Ruminococcus* abundance, only RH19 showed a significant decrease (*P* < 0.05), with no significant differences observed among other treatments (*P* > 0.05). Notably, *Candidatus*_*Cryptobacteroides* abundance in RH27 was significantly higher than in all other treatment groups (*P* < 0.05). At the species level, RH3, RH14, and RH19 had higher *Prevotella*_sp. abundance than NC, while RH27 and RH35 had lower (*P* < 0.05). The RH19 treatment group demonstrated a significantly higher abundance of *M. elsdenii* in the fermentation broth compared to other treatments (*P* < 0.05, Fig. S6A). The archaeal community was dominated by the phylum Euryarchaeota (91.60% ± 1.06%) and the genus *Methanobrevibacter* (80.92% ± 2.38%) (Fig. 2F). No significant differences were found at the phylum or genus level (*P* > 0.05), but a downward trend in the abundance of Euryarchaeota and *Methanobrevibacter* was observed in the supplemented groups (Fig. 3B).

The abundance profiles and differential analysis of eukaryotic species after supplementation with *Prevotella* and *Megasphaera elsdenii* were evaluated. At the phylum level, Ciliophora was the most abundant group, with no significant differences observed among treatments (*P* > 0.05). In contrast, the fungal phylum Mucoromycota was the only group significantly reduced following supplementation (*P* < 0.05). At the genus level, no significant differences in abundance were detected. At the species level, *Blepharisma stoltei* was significantly decreased in the RH3 and RH27 groups compared with the NC group (*P* < 0.05), whereas no significant changes were observed in other eukaryotic species (Fig. S5).

Since enteric methane in dairy cows is primarily produced by archaea, and bacteria—which dominate the rumen microbiota—generate the key substrates for methanogenesis (e.g., H_2_, CO_2_, VFAs, and methyl compounds), only Bacteria and Archaea were included in subsequent comparative analyses [40].

### Functional profiling of the rumen microbiota

Functional profiling of the rumen microbiota was conducted through annotation of metagenomic sequences against KEGG pathways and CAZyme genes.

Sequence mapping identified 321 KEGG level-3 pathways representing core rumen metabolic functions. Among the top 50 differentially abundant pathways (Fig. 3C), the relative abundance of methane metabolism-related pathways was significantly reduced in all treatments compared with the NC group (*P* < 0.05). Within metabolic pathways, only the RH27 group showed no difference from the control (*P* > 0.05), whereas the other treatments showed significantly lower relative abundances of methane metabolism-related pathways (*P* < 0.05).

**Fig. 4:**
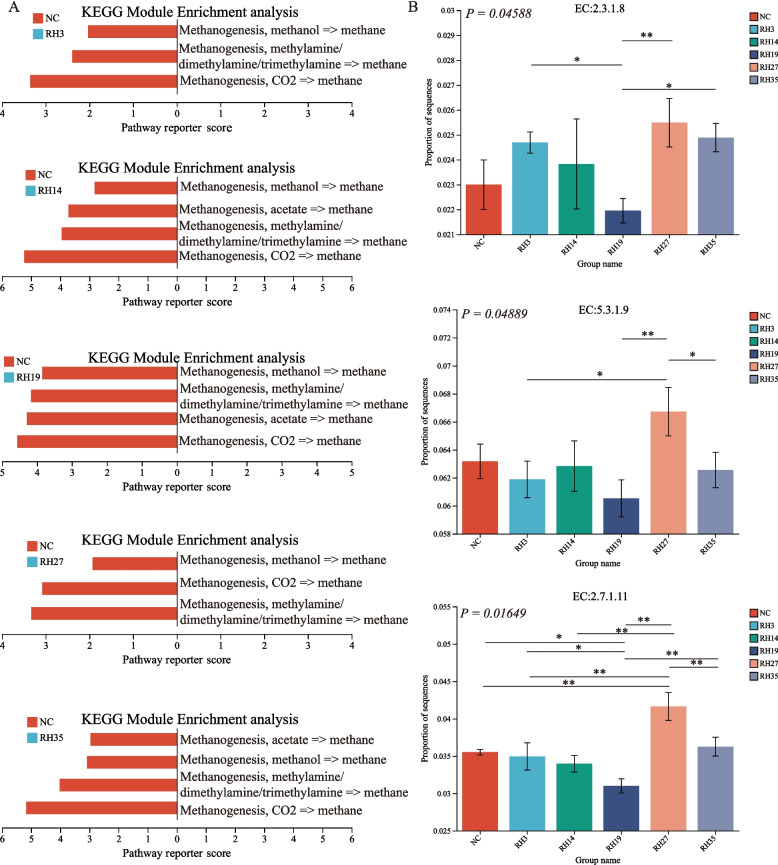
Functional differences in methanogenesis-related pathways and KEGG Orthology (KO) enzymes across different treatments in an in vitro rumen fermentation system. **A** Comparison of methanogenesis-related KEGG modules among different treatment groups. The pathways include methanogenesis from methanol, methylamine/dimethylamine/trimethylamine, acetate, and CO_2_ reduction to methane. The bar lengths represent the relative abundance of each KEGG module. **B** Differential abundance analysis of KEGG Orthology (KO) enzymes involved in methanogenesis across treatments. The bar plots show the relative abundance of KO enzymes in each group (NC, RH13, RH14, RH19, RH27, RH35), with error bars indicating standard deviations. Statistical significance between groups is indicated by asterisks (^*^P < 0.05, ^**^P < 0.01), and exact *P*-values are provided where applicable. Different colors represent different treatment groups

Key pathways related to amino acid, carbohydrate, lipid, and energy metabolism were further examined due to their link with enteric methane emissions and energy use (Fig. S6B–E). In amino acid metabolism, six pathways showed significant differences in relative abundance (*P* < 0.05). For lipid metabolism, only three pathways differed significantly in relative abundance (*P* < 0.05). In energy metabolism, only Carbon fixation by Calvin cycle showed a significant difference in relative abundance (*P* < 0.05). KEGG module analysis revealed that among the top 50 modules. The cobalamin (vitamin B_12_) biosynthesis module remained unchanged in RH19 but was downregulated in all other treatments (*P* < 0.05). Additionally, the acetoclastic methanogenesis pathway was significantly downregulated in RH14, RH19, and RH35 (*P* < 0.05) (Fig. 3D). However, given the minor role of acetoclastic methanogenesis in the rumen ecosystem, its downregulation, while statistically significant, likely contributes less to the overall methane mitigation [41, 42]. More importantly, differential analysis of other core methanogenic modules showed that the carbon dioxide reduction to methane module—the dominant hydrogenotrophic pathway in the rumen—along with the methylamine and methanol methanogenesis modules, were significantly enriched in the NC group compared to the supplemented treatments (*P* < 0.05) (Fig. 4A) [43, 44].

A total of 613 CAZyme-encoding genes were identified, including 17 auxiliary activities (AAs), 77 carbohydrate-binding modules (CBMs), 17 carbohydrate esterases (CEs), 338 glycoside hydrolases (GHs), 92 glycosyltransferases (GTs), and 71 polysaccharide lyases (PLs).

To explore the metabolic mechanisms underlying fermentation shifts, key enzymes involved in glucose conversion to acetate, propionate, and butyrate were first examined (Fig. S6F). A total of 14 enzymes associated with these pathways were identified. Among them, the abundances of EC:5.3.1.9 and EC:2.7.1.11 were significantly higher in the RH27 group compared to other treatments (*P* < 0.05; Fig. 4B). However, the metagenomic data did not allow resolution of whether these enzymes were associated with microorganisms involved in hydrogen-producing or propionate-associated fermentation pathways. Notably, propionate formation serves as a major sink for metabolic hydrogen, thereby limiting the accumulation of molecular H₂, a key substrate for methanogenesis. This suggests that enhanced propionate production may indirectly contribute to reduced methane emissions.

To support these functional shifts, CAZyme profiles in the rumen microbiome were further analyzed. At the community-wide level, total CAZyme abundance differed among groups (Fig. S7A), with RH35 showing higher abundance than NC and RH14 (*P* < 0.05), and RH27 higher than RH14 (*P* < 0.05). However, no significant differences were observed in the abundance of individual CAZyme classes (AA, CBM, CE, GH, GT, and PL) across treatments (Fig. S7B).

Further analysis within each CAZyme class revealed specific responses to supplementation with *Prevotella* and *Megasphaera elsdenii*. Significant differences (*P* < 0.05) were detected in 2 AAs, 9 CBMs, 40 GHs, 5 CEs, 8 GTs, and 1 PL (Fig. S7C–F). To further characterize carbohydrate degradation capacity, CAZyme genes encoding cellulases, hemicellulases, and ligninases were analyzed (Fig. S7G; Table S5). Among 64 GH families examined, only seven (GH92, GH67, GH39, GH1, GH4, GH38, and GH113) showed significant differences in abundance among treatments (*P* < 0.05).

### Microbial interactions and correlations with fermentation parameters

**Fig. 5:**
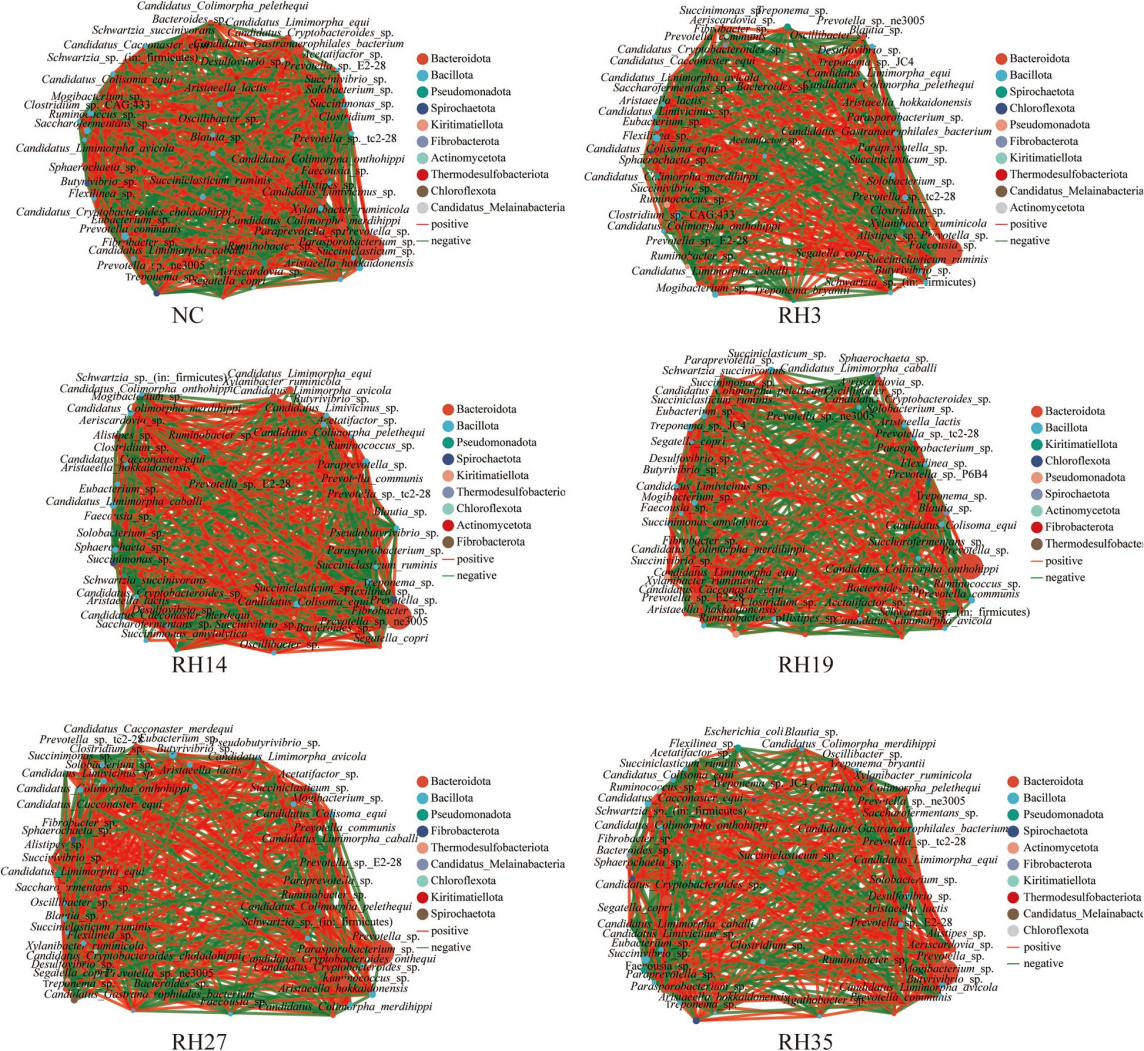
Co-occurrence networks of rumen bacterial communities following supplementation with *Prevotella* and *Megasphaera elsdenii*. The networks were constructed based on Spearman’s rank correlation analysis among bacterial taxa. Only significant correlations (*P* < 0.05) are displayed. Red edges indicate positive correlations, whereas green edges represent negative correlations. Node size is proportional to the mean relative abundance of each taxon. Different panels correspond to distinct treatment groups. Nodes represent bacterial taxa at the genus level

**Fig. 6:**
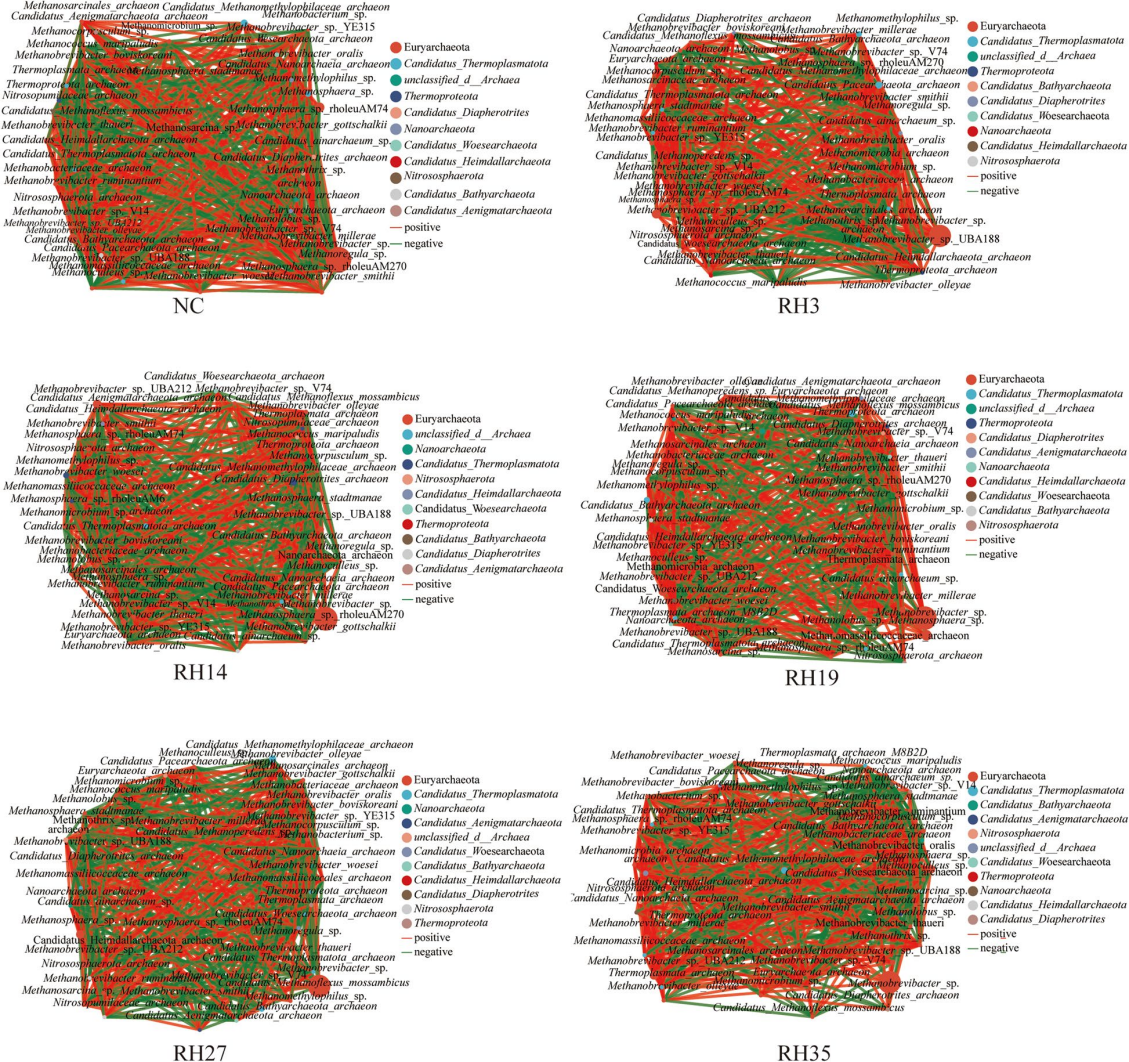
Co-occurrence networks of rumen archaeal communities in response to supplementation with *Prevotella* and *Megasphaera elsdenii*. The networks were constructed based on Spearman’s rank correlation analysis among archaeal taxa. Only significant correlations (*P* < 0.05) are displayed. Red edges indicate positive correlations, whereas green edges represent negative correlations. Node size is proportional to the mean relative abundance of each taxon. Different panels correspond to distinct treatment groups. Nodes represent archaeal taxa at the genus level, and node colors denote their taxonomic classification at the phylum level

Co-occurrence network analysis uncovered potential interactions among microbes. Bacterial network analysis identified a total of 5,342 significant associations, with distinct structures for each treatment (Fig. 5). The most frequent negative correlations were found between the Bacteroidetes and Bacillota phyla. Archaeal co-occurrence network analysis identified 5,558 significant relationships, with the most prevalent positive correlations occurring within the Euryarchaeota phylum (Fig. 6). Supplementation with rumen-derived bacteria reduced interconnectivity within the archaeal network.

**Fig. 7:**
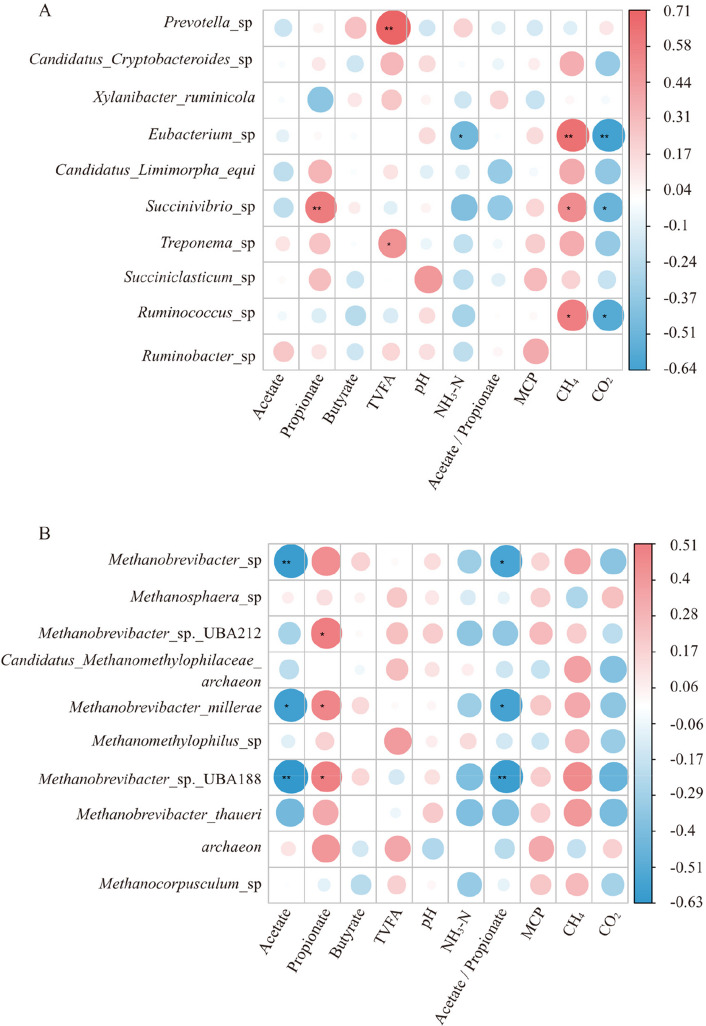
Correlation analysis between rumen microbial taxa and fermentation parameters. **A** Correlation heatmap showing relationships between dominant bacterial genera and rumen fermentation parameters. **B** Correlation heatmap showing relationships between dominant archaeal genera and rumen fermentation parameters. Correlations were calculated using Spearman’s rank correlation analysis. Circle color indicates the direction of the correlation, with red representing positive correlations and blue representing negative correlations. Circle size is proportional to the absolute value of the correlation coefficient, reflecting the strength of the association. Asterisks indicate statistically significant correlations (^*^P < 0.05, ^**^P < 0.01). The fermentation parameters include acetate, propionate, butyrate, total volatile fatty acids (TVFA), pH, ammonia nitrogen (NH_3_-N), acetate-to-propionate ratio (Acetate/Propionate), microbial crude protein (MCP), methane (CH_4_), and carbon dioxide (CO_2_). Each row represents a microbial taxon at the genus level, and each column represents a fermentation parameter. The heatmaps illustrate the potential functional associations between rumen microbial communities and fermentation characteristics under different treatments

Correlation analysis revealed significant associations between microbial taxa and fermentation parameters. Among bacterial genera, *Prevotella_*sp*.* showed a significant positive correlation with TVFA (*P* < 0.05). *Eubacterium_*sp*.* was positively correlated with CH_4_ production (*P* < 0.05). In addition, *Succinivibrio_*sp*.* exhibited a significant positive correlation with propionate, while *Ruminococcus_*sp*.* showed a positive correlation with CH_4_ but a negative correlation with CO_2_ (*P* < 0.05), indicating their potential roles in rumen fermentation and gas metabolism (Fig. 7A).

Among archaeal taxa, *Methanobrevibacter_*sp*.* displayed significant negative correlations with NH_3_-N and acetate (*P* < 0.05). Furthermore, *Methanobrevibacter*_sp._UBA188 and *Methanobrevibacter_millerae* were negatively associated with acetate and NH_3_-N but positively correlated with CH_4_ production (*P* < 0.05). These results suggest that methanogenic archaea are closely linked to nitrogen utilization and methane emissions in the rumen ecosystem (Fig. 7B).

## Discussion

### Genomic characteristics of isolated strains

This study successfully isolated and characterized four *Prevotella* strains (RH3, RH14, RH27, RH35) and one *Megasphaera elsdenii* strain (RH19) from the rumen. Their functional roles in rumen fermentation and methane mitigation were systematically evaluated through genomic annotation, in vitro fermentation, and metagenomic analyses. The functional roles of these isolates in rumen fermentation are rooted in their distinct genomic architectures [45]. RH3 had larger genome and higher proportion of CEs compared to other *Prevotella* strains suggested an expanded carbohydrate esterification capacity [46]. In contrast, *M. elsdenii* RH19 showed relatively low PL abundance but comparatively high GT abundance. Glycosyltransferases are mainly involved in glycan biosynthesis and carbohydrate processing, reflecting general carbohydrate metabolic potential rather than pathway-specific enzymes. This is consistent with genome-based analyses showing that *M. elsdenii* possesses a broad capacity for carbohydrate utilization [47]. The relatively high GH abundance observed in *Prevotella* strains is consistent with previous reports describing the extensive glycoside hydrolase repertoires and plant polysaccharide degradation potential of ruminal *Prevotella* species [48].

### Strain-specific effects on rumen fermentation and methane production

The observed in vitro rumen fermentation dynamics indicate that methane responses were strain- and time-dependent rather than uniformly reduced by all treatments. At 12 h, all supplemented groups showed higher CH_4_ production than the control, likely reflecting increased overall fermentation activity, as supported by elevated gas and CO_2_ production.

At later stages, selective mitigation effects became evident. RH14 reduced methane production at both 24 and 48 h, while RH35 showed a reduction at 48 h, indicating strain-specific effects on rumen fermentation. The lower acetate-to-propionate ratio observed at specific time points (e.g., RH14 at 48 h) may indicate a transient shift in fermentation patterns toward propionate-associated pathways, which are theoretically linked to metabolic hydrogen utilization and reduced H₂ availability for hydrogenotrophic methanogenesis [49, 50]. However, these shifts were not consistently observed across all strains or incubation stages. Overall, methane mitigation appeared to depend on fermentation stage and microbial characteristics rather than representing a uniform response among all supplemented strains. Interestingly, the higher TVFA concentration observed at 48 h followed by a decline at 72 h may be associated with reduced availability of fermentable substrates during the late incubation stage as well as potential shifts in microbial metabolic activity, including possible secondary fermentation processes involving VFAs [51, 52]. The increased valerate concentration observed in several treatments at 72 h suggests that part of the fermentation intermediates may have undergone additional metabolic conversion. However, these interpretations are speculative, as such metabolic pathways were not directly investigated in this study. As fermentation progresses, reduced availability of fermentable substrates and changes in microbial activity lead to decreased VFA production, a pattern commonly observed in in vitro rumen fermentation systems [27, 49].

### Microbial community shifts associated with methane mitigation

Metagenomic analysis provides insights into the potential ecological and functional mechanisms underlying methane reduction. The observed increase in Bacteroidetes (particularly *Prevotella*) and decrease in Bacillota (e.g., *Ruminococcus*) in treatments such as RH3 and RH19 suggested niche competition for fibrous substrates. This competition may reduce the availability of H_2_ for hydrogenotrophic methanogenesis [53, 54]. The enrichment of *Candidatus_Cryptobacteroides*, a putative propionate-producing bacterium, in RH27 may further contribute to reduced methanogenesis by acting as a sink for metabolic hydrogen, thereby indirectly limiting H_2_ availability for hydrogenotrophic methanogens [53–55]. Following in vitro supplementation, the RH19 group showed a higher relative abundance of *Megasphaera elsdenii*, indicating its establishment under in vitro conditions. Interestingly, *Prevotella* reached its highest abundance in the *M. elsdenii*-supplemented treatment rather than in the *Prevotella*-supplemented groups. This pattern may reflect indirect ecological interactions within the rumen microbiome. One possible explanation is metabolic cross-feeding, whereby *Prevotella*_sp. produce lactate and other fermentation intermediates that can subsequently be utilized by *M. elsdenii*. By utilizing lactate and altering fermentation patterns, *M. elsdenii* may indirectly modify the ruminal environment in ways that favor the persistence or relative abundance of *Prevotella*_sp. In addition, the reduction of lactate accumulation and improved ruminal pH stability associated with *M. elsdenii* activity may further contribute to these community shifts [56, 57]. Given that *M. elsdenii* typically occurs at low abundance in the rumen (usually below 1%) [58], it was not detected among dominant taxa in the initial community analysis. These observations highlight the potential importance of indirect microbial interactions in shaping community structure and function, although further studies are required to experimentally validate these mechanisms.

### Functional metagenomic responses related to methanogenesis

The downward trend in the hydrogenotrophic genus *Methanobrevibacter*, together with the reduced relative abundance of the dominant “carbon dioxide reduction to methane” pathway, suggests that methane mitigation may have been associated with altered hydrogen metabolism rather than elimination of methanogens [40, 44, 59, 60]. The significant downregulation of the acetoclastic pathway, while statistically significant, likely contributes less to overall mitigation given its minor role in the rumen [41, 42]. More importantly, the reduced abundance of the dominant hydrogenotrophic CO_2_-reduction pathway in supplemented groups supports the possibility that bacterial supplementation influenced metabolic pathways associated with hydrogen utilization [40]. However, direct measurements of H_2_ flux and metabolic hydrogen redistribution were not performed in the present study. Therefore, the proposed mechanisms remain inferential and should be interpreted cautiously.

Furthermore, the downregulation of the cobalamin (vitamin B12) biosynthesis module in most treatments may also have contributed to reduced methanogenic activity, because vitamin B12 serves as an important coenzyme in several methanogenesis-related reactions [61, 62]. Nevertheless, the direct relationship between reduced cobalamin biosynthesis and methane suppression was not experimentally validated in this study.

The shift in specific fiber-degrading CAZyme families may also influence fermentation end-products and associated hydrogen metabolism [63]. The upregulation of key glycolytic enzymes (EC:5.3.1.9 and EC:2.7.1.11) in the RH27 treatment suggests enhanced glycolytic flux through the Embden–Meyerhof–Parnas (EMP) pathway, leading to increased pyruvate formation. Because pyruvate serves as a central metabolic intermediate, it may subsequently enter multiple downstream fermentation pathways, including acetate- and propionate-associated metabolism. However, the metagenomic data did not allow resolution of whether these enzymes were specifically associated with hydrogen-producing or propionate-associated fermentative microorganisms. Therefore, although these findings may be consistent with altered fermentation pathways linked to metabolic hydrogen utilization, the specific microbial contributors remain unresolved.

### Microbial interaction networks and ecological implications

The microbial interaction networks and correlation analyses integrate these findings. The elevated Bacteroidetes-to-Bacillota ratio in supplemented groups is significant, as previous research correlates an increased ratio with enhanced energy utilization efficiency in dairy cows [64, 65]. This suggests that supplementation of rumen microorganisms may improve feed energy utilization efficiency, as reflected by shifts in fermentation patterns toward increased VFA production and reduced energy loss as methane. The reduced interconnectivity within the archaeal network upon bacterial supplementation may indicate a decline in methanogenic activity and a shift in microbial interactions that are less favorable for dominant methanogenic populations [50, 66]. The significant positive correlation between *Prevotella*_sp. and TVFA is consistent with previous reports linking its enrichment to enhanced VFA production [67]. The positive correlation between *Eubacterium* sp. and methane should be interpreted with caution, as members of this genus exhibit diverse metabolic roles, including both H₂-producing fermentation and H₂-consuming acetogenesis [68]. Given that methanogenesis primarily depends on H₂ and formate availability and often outcompetes acetogenesis under rumen conditions [69], this relationship may reflect substrate availability rather than a direct causal contribution to methane production.

## Conclusions

In conclusion, supplementation with rumen-derived *Prevotella* and *M. elsdenii* strains influenced rumen fermentation patterns, microbial community structure, and methanogenesis-related pathways under in vitro conditions. Certain strains, particularly RH14 and RH35, reduced methane production during specific incubation stages, accompanied by changes in fermentation characteristics and lower relative abundances of key hydrogenotrophic methanogenesis pathways. However, several proposed mechanisms, including metabolic hydrogen redistribution and microbial cross-feeding interactions, remain inferential and require further experimental validation.

## Supplementary Information

Additional file 1: Table S1. Composition and nutritional level of diets for dairy cows. Table S2. Substrates fermentable by *Prevotella* and *Megasphaera elsdenii*. Table S3. Genomic characteristics of *Prevotella* and *Megasphaera elsdenii*. Table S4. Summary of metagenomic sequencing data from in vitro rumen fermentation samples. Table S5. The relative abundances of GH family genes encoded fibrolytic enzymes. Fig. S1. Morphological, Gram-staining, and genomic features of the isolated *Prevotella* and *Megasphaera elsdenii* strains. Fig. S2. CAZyme gene counts in *Prevotella* and *Megasphaera elsdenii* strains. Fig. S3. Functional genome annotation of *Prevotella* and *Megasphaera elsdenii* based on KEGG and COG databases. Fig. S4. Time-resolved profiles of volatile fatty acids (VFAs) and total VFA-carbon during in vitro rumen fermentation following supplementation with rumen-derived bacterial strains. Fig. S5. Eukaryotic community composition and differential analysis in rumen fermentation samples. Fig. S6. Differential abundance of *Megasphaera elsdenii* and functional metabolic features based on metagenomic analysis in an in vitro dairy cow fermentation system. Fig. S7. Comparative analysis of carbohydrate-active enzymes (CAZymes) in the rumen microbiome of dairy cows based on metagenomic sequencing.

## Data Availability

The nucleotide sequences of the 16S rRNA gene from five strains of rumen bacteria isolated from dairy cows have been submitted to GenBank and assigned the following accession numbers: strain RH3 (accession number: PX426733), strain RH14 (PX426734), strain RH19 (PX426735), strain RH27 (PX426736), and strain RH35 (PX426737). The rumen metagenome sequences and bacterial whole-genome sequences were deposited into NCBI Sequence Read Archive (SRA) under the accession number of PRJNA1312568 and PRJNA1314459.

## Abbreviations

CH_4_: Methane
CO_2_: Carbon dioxide
GC: Guanine cytosine
H_2_: Hydrogen
MCP: Microbial crude protein
NH_3_-N: Ammonia nitrogen
OD: Optical density
VFA: Volatile fatty acid
TVFA: Total volatile fatty acids

## References

[1] Filonchyk M, Peterson MP, Zhang L, Hurynovich V, He Y. Greenhouse gases emissions and global climate change: examining the influence of CO_2_, CH_4_, and N_2_O. Sci Total Environ. 2024;935:173359. 10.1016/j.scitotenv.2024.173359. 38768722 10.1016/j.scitotenv.2024.173359

[2] Anderson B, Bartlett KB, Frolking S, Hayhoe K, Jenkins JC, Salas WA. Methane and nitrous oxide emissions from natural sources. Washington DC: Office of Atmospheric Programs, US EPA, EPA 430-R-10-001; 2010.

[3] Steinfeld H. Livestock’s long shadow: environmental issues and options. Rome: FAO; 2006.

[4] Gerber PJ, Steinfeld H, Henderson B, Mottet A, Opio C, Dijkman J, et al. Tackling climate change through livestock: a global assessment of emissions and mitigation opportunities. Rome: Food and Agriculture Organization of the United Nations (FAO); 2013.

[5] Bergman E. Energy contributions of volatile fatty acids from the gastrointestinal tract in various species. Physiol Rev. 1990;70:567–90. 10.1152/physrev.1990.70.2.567. 2181501 10.1152/physrev.1990.70.2.567

[6] Vijn S, Compart DP, Dutta N, Foukis A, Hess M, Hristov AN, et al. Key considerations for the use of seaweed to reduce enteric methane emissions from cattle. Front Vet Sci. 2020;7:597430. 10.3389/fvets.2020.597430. 33426018 10.3389/fvets.2020.597430 PMC7785520

[7] Palangi V, Taghizadeh A, Abachi S, Lackner M. Strategies to mitigate enteric methane emissions in ruminants: a review. Sustainability. 2022;14:13229. 10.3390/su142013229.

[8] Wanapat M, Cherdthong A, Phesatcha K, Kang S. Dietary sources and their effects on animal production and environmental sustainability. Anim Nutr. 2015;1:96–103. 10.1016/j.aninu.2015.07.004. 29767156 10.1016/j.aninu.2015.07.004 PMC5945976

[9] Jeyanathan J, Martin C, Morgavi D. The use of direct-fed microbials for mitigation of ruminant methane emissions: a review. Animal. 2014;8:250–61. 10.1017/S1751731113002085. 24274095 10.1017/S1751731113002085

[10] Aguilar-Marin SB, Betancur-Murillo CL, Isaza GA, Mesa H, Jovel J. Lower methane emissions were associated with higher abundance of ruminal *Prevotella* in a cohort of Colombian buffalos. BMC Microbiol. 2020;20:364. 10.1186/s12866-020-02037-6. 33246412 10.1186/s12866-020-02037-6 PMC7694292

[11] Dodd D, Moon YH, Swaminathan K, Mackie RI, Cann IK. Transcriptomic analyses of xylan degradation by *Prevotella bryantii* and insights into energy acquisition by xylanolytic Bacteroidetes. J Biol Chem. 2010;285:30261–73. 10.1074/jbc.M110.141788. 20622018 10.1074/jbc.M110.141788 PMC2943253

[12] Kabel MA, Yeoman CJ, Han Y, Dodd D, Abbas CA, de Bont JA, et al. Biochemical characterization and relative expression levels of multiple carbohydrate esterases of the xylanolytic rumen bacterium *Prevotella ruminicola *23 grown on an ester-enriched substrate. Appl Environ Microbiol. 2011;77:5671–81. 10.1128/AEM.05321-11. 21742923 10.1128/AEM.05321-11 PMC3165261

[13] Louis P, Flint HJ. Formation of propionate and butyrate by the human colonic microbiota. Environ Microbiol. 2017;19:29–41. 10.1111/1462-2920.13589. 27928878 10.1111/1462-2920.13589

[14] Wu QC, Wang WK, Zhang F, Li WJ, Wang YL, Lv LK, et al. Dietary cysteamine supplementation remarkably increased feed efficiency and shifted rumen fermentation toward glucogenic propionate production via enrichment of *Prevotella* in feedlot lambs. Microorganisms. 2022;10:1105. 10.3390/microorganisms10061105. 35744623 10.3390/microorganisms10061105 PMC9227252

[15] Trautmann A, Schleicher L, Koch A, Gunther J, Steuber J, Seifert J. A shift towards succinate-producing *Prevotella* in the ruminal microbiome challenged with monensin. Proteomics. 2023;23:2200121. 10.1002/pmic.202200121. 10.1002/pmic.202200121 36444514

[16] Strobel HJ. Vitamin B12-dependent propionate production by the ruminal bacterium *Prevotella ruminicola *23. Appl Environ Microbiol. 1992;58:2331–3. 10.1128/aem.58.7.2331-2333.1992. 1637169 10.1128/aem.58.7.2331-2333.1992 PMC195777

[17] Kittelmann S, Pinares-Patiño CS, Seedorf H, Kirk MR, Ganesh S, McEwan JC, et al. Two different bacterial community types are linked with the low-methane emission trait in sheep. PLoS One. 2014;9(7):e103171. 10.1371/journal.pone.0103171. 10.1371/journal.pone.0103171 PMC4117531 25078564

[18] Counotte GHM. Regulation of lactate metabolism in the rumen. Wageningen: Wageningen University; 1981. 10.1007/BF02214975. 10.1007/BF02214975 7048723

[19] Hobson PN, Stewart CS. The rumen microbial ecosystem. Dordrecht: Springer; 1988. 10.1007/978-94-009-1453-7.

[20] Prabhu R, Altman E, Eiteman MA. Lactate and acrylate metabolism by *Megasphaera elsdenii* under batch and steady-state conditions. Appl Environ Microbiol. 2012;78:8564–70. 10.1128/AEM.02443-12. 23023753 10.1128/AEM.02443-12 PMC3502912

[21] Bolger AM, Lohse M, Usadel B. Trimmomatic: a flexible trimmer for Illumina sequence data. Bioinformatics. 2014;30:2114–20. 10.1093/bioinformatics/btu170. 24695404 10.1093/bioinformatics/btu170 PMC4103590

[22] Simpson JT, Wong K, Jackman SD, Schein JE, Jones SJM, Birol I. ABySS: a parallel assembler for short read sequence data. Genome Res. 2009;19:1117–23. 10.1101/gr.089532.108. 19251739 10.1101/gr.089532.108 PMC2694472

[23] Xu M, Guo L, Gu S, Wang O, Zhang R, Peters BA, et al. TGS-GapCloser: a fast and accurate gap closer for large genomes with low coverage of error-prone long reads. Gigascience. 2020;9:giaa094. 10.1093/gigascience/giaa094. 32893860 10.1093/gigascience/giaa094 PMC7476103

[24] Besemer J, Borodovsky M. GeneMark: web software for gene finding in prokaryotes, eukaryotes and viruses. Nucleic Acids Res. 2005;33:W451–4. 10.1093/nar/gki487. 15980510 10.1093/nar/gki487 PMC1160247

[25] Chan PP, Lowe TM. tRNAscan-SE: searching for tRNA genes in genomic sequences. In: Kollmar M, editor. Gene Prediction. Methods in Molecular Biology, vol. 1962. New York: Humana; 2019. 10.1007/978-1-4939-9173-0_1. 10.1007/978-1-4939-9173-0_1 PMC6768409 31020551

[26] Lagesen K, Hallin P, Rodland EA, Staerfeldt HH, Rognes T, Ussery DW. RNAmmer: consistent and rapid annotation of ribosomal RNA genes. Nucleic Acids Res. 2007;35:3100–8. 10.1093/nar/gkm160. 17452365 10.1093/nar/gkm160 PMC1888812

[27] Menke KH, Raab L, Salewski A, Steingass H, Fritz D, Schneider W. The estimation of the digestibility and metabolizable energy content of ruminant feedingstuffs from the gas production when they are incubated with rumen liquor in vitro. J Agric Sci. 1979;93:217–22. 10.1017/S0021859600086305.

[28] Susanto I, Wiryawan KG, Suharti S, Retnani Y, Zahera R, Jayanegara A. Evaluation of *Megasphaera elsdenii* supplementation on rumen fermentation, production performance, carcass traits and health of ruminants: a meta-analysis. Anim Biosci. 2023;36:879. 10.5713/ab.22.0258. 36634661 10.5713/ab.22.0258 PMC10164541

[29] Weatherburn MW. Phenol-hypochlorite reaction for determination of ammonia. Anal Chem. 1967;39:971–4. 10.1021/ac60252a045.

[30] Zinn RA, Owens FN. A rapid procedure for purine measurement and its use for estimating net ruminal protein synthesis. Can J Anim Sci. 1986;66:157–66. 10.4141/cjas86-017.

[31] Erwin ES, Marco GJ, Emery EM. Volatile fatty acid analyses of blood and rumen fluid by gas chromatography. J Dairy Sci. 1961;44:1768–71. 10.3168/jds.S0022-0302(61)89956-6.

[32] Chen S, Zhou Y, Chen Y, Gu J. fastp: an ultra-fast all-in-one FASTQ preprocessor. Bioinformatics. 2018;34:i884–90. 10.1093/bioinformatics/bty560. 30423086 10.1093/bioinformatics/bty560 PMC6129281

[33] Li H, Durbin R. Fast and accurate short read alignment with Burrows–Wheeler transform. Bioinformatics. 2009;25:1754–60. 10.1093/bioinformatics/btp324. 19451168 10.1093/bioinformatics/btp324 PMC2705234

[34] Li D, Liu CM, Luo R, Sadakane K, Lam TW. MEGAHIT: an ultra-fast single-node solution for large and complex metagenomics assembly via succinct de Bruijn graph. Bioinformatics. 2015;31:1674–6. 10.1093/bioinformatics/btv033. 25609793 10.1093/bioinformatics/btv033

[35] Hyatt D, Chen GL, LoCascio PF, Land ML, Larimer FW, Hauser LJ. Prodigal: prokaryotic gene recognition and translation initiation site identification. BMC Bioinformatics. 2010;11:119. 10.1186/1471-2105-11-119. 20211023 10.1186/1471-2105-11-119 PMC2848648

[36] Noguchi H, Park J, Takagi T. MetaGene: prokaryotic gene finding from environmental genome shotgun sequences. Nucleic Acids Res. 2006;34:5623–30. 10.1093/nar/gkl723. 17028096 10.1093/nar/gkl723 PMC1636498

[37] Fu L, Niu B, Zhu Z, Wu S, Li W. CD-HIT: accelerated for clustering the next-generation sequencing data. Bioinformatics. 2012;28:3150–2. 10.1093/bioinformatics/bts565. 23060610 10.1093/bioinformatics/bts565 PMC3516142

[38] Li R, Li Y, Kristiansen K, Wang J. SOAP: short oligonucleotide alignment program. Bioinformatics. 2008;24:713–4. 10.1093/bioinformatics/btn025. 18227114 10.1093/bioinformatics/btn025

[39] Buchfink B, Xie C, Huson DH. Fast and sensitive protein alignment using DIAMOND. Nat Methods. 2015;12:59–60. 10.1038/nmeth.3176. 25402007 10.1038/nmeth.3176

[40] Hook SE, Wright ADG, McBride BW. Methanogens: methane producers of the rumen and mitigation strategies. Archaea. 2010;2010:945785. 10.1155/2010/945785. 21253540 10.1155/2010/945785 PMC3021854

[41] Liu Y, Whitman WB. Metabolic, phylogenetic, and ecological diversity of the methanogenic archaea. Ann N Y Acad Sci. 2008;1125:171–89. 10.1196/annals.1419.019. 18378594 10.1196/annals.1419.019

[42] Patra AK, Park T, Kim M, Yu Z. Rumen methanogens and mitigation of methane emission by anti-methanogenic compounds and substances. J Anim Sci Biotechnol. 2017;8:13. 10.1186/s40104-017-0145-9. 28149512 10.1186/s40104-017-0145-9 PMC5270371

[43] Hook SE. Structure of the archaeal community of the rumen. Appl Environ Microbiol. 2008;74:3619–25. 10.1128/AEM.02812-07. 18424540 10.1128/AEM.02812-07 PMC2446570

[44] Danielsson R, Dicksved J, Sun L, Gonda H, Muller B, Schnurer A, et al. Methane production in dairy cows correlates with rumen methanogenic and bacterial community structure. Front Microbiol. 2017;8:226. 10.3389/fmicb.2017.00226. 28261182 10.3389/fmicb.2017.00226 PMC5313486

[45] Accetto T, Avgustin G. Polysaccharide utilization locus and CAZyme genome repertoires reveal diverse ecological adaptation of *Prevotella* species. Syst Appl Microbiol. 2015;38:453–61. 10.1016/j.syapm.2015.07.007. 26415759 10.1016/j.syapm.2015.07.007

[46] Dodd D, Mackie RI, Cann IK. Xylan degradation, a metabolic property shared by rumen and human colonic Bacteroidetes. Mol Microbiol. 2011;79:292–304. 10.1111/j.1365-2958.2010.07473.x. 21219452 10.1111/j.1365-2958.2010.07473.x PMC4561535

[47] Marx H, Graf AB, Tatto NE, Thallinger GG, Mattanovich D, Sauer M. Genome sequence of the ruminal bacterium *Megasphaera elsdenii*. J Bacteriol. 2011;193:557–8. 10.1128/jb.05861-11. 10.1128/JB.05861-11 PMC3187434 21914887

[48] Accetto T, Avguštin G. The diverse and extensive plant polysaccharide degradative apparatuses of the rumen and hindgut *Prevotella* species: a factor in their ubiquity? Syst Appl Microbiol. 2019;42:107–16. 10.1016/j.syapm.2018.10.001. 30853065 10.1016/j.syapm.2018.10.001

[49] Janssen PH. Influence of hydrogen on rumen methane formation and fermentation balances through microbial growth kinetics and fermentation thermodynamics. Anim Feed Sci Technol. 2010;160:1–22. 10.1016/j.anifeedsci.2010.07.002. Get rights and content.

[50] Ungerfeld EM. Metabolic hydrogen flows in rumen fermentation: principles and possibilities of interventions. Front Microbiol. 2020;11:589. 10.3389/fmicb.2020.00589. 32351469 10.3389/fmicb.2020.00589 PMC7174568

[51] Bevilacqua A, Regueira A, Mauricio-Iglesias M, Lema JM, Carballa M. Chain elongation may occur in protein mixed-culture fermentation without supplementing electron donor compounds. J Environ Chem Eng. 2022;10:106943. 10.1016/j.jece.2021.106943.

[52] Li MM, Ghimire S, Wenner BA, Bradford BJ, Drouillard JS, Nagaraja TG, et al. Effects of acetate, propionate, and pH on volatile fatty acid thermodynamics in continuous cultures of ruminal contents. J Dairy Sci. 2022;105:8879–97. 10.3168/jds.2022-22080. 36085109 10.3168/jds.2022-22084

[53] Purushe J, Fouts DE, Morrison M, White BA, Mackie RI, Coutinho PM, et al. Comparative genome analysis of *Prevotella ruminicola* and *Prevotella bryantii*: insights into their environmental niche. Microb Ecol. 2010;60:721–9. 10.1007/s00248-010-9692-8. 20585943 10.1007/s00248-010-9692-8

[54] McAllister TA, Beauchemin KA, Alazzeh AY, Baah J, Teather RM, Stanford K. The use of direct fed microbials to mitigate pathogens and enhance production in cattle. Can J Anim Sci. 2011;91:193–211. 10.4141/cjas10047.

[55] Dirks B, Davis TL, Carnero EA, Corbin KD, Smith SR, Rittmann BE, et al. Methanogenesis associated with altered microbial production of short-chain fatty acids and human-host metabolizable energy. ISME J. 2025;19:wraf103. 10.1093/ismejo/wraf103. 40403748 10.1093/ismejo/wraf103 PMC12156016

[56] Cabral LS, Weimer PJ. *Megasphaera elsdenii*: its role in ruminant nutrition and its potential industrial application for organic acid biosynthesis. Microorganisms. 2024;12:219. 10.3390/microorganisms12010219. 38276203 10.3390/microorganisms12010219 PMC10819428

[57] Monteiro HF, Agustinho BC, Vinyard JR, Harden T, Bennett SL, ArceCordero JA, et al. *Megasphaera elsdenii* and *Saccharomyces cerevisiae* as direct-fed microbials during an in vitro acute ruminal acidosis challenge. Sci Rep. 2022;12:7978. 10.1038/s41598-022-11959-2. 35562415 10.1038/s41598-022-11959-2 PMC9106753

[58] Rico DE, Preston SH, Risser JM, Harvatine KJ. Rapid changes in key ruminal microbial populations during the induction of and recovery from diet-induced milk fat depression in dairy cows. Br J Nutr. 2015;114:358–67. 10.1017/S0007114515001865. 26123320 10.1017/S0007114515001865

[59] Leahy SC, Kelly WJ, Altermann E, Ronimus RS, Yeoman CJ, Pacheco DM, et al. The genome sequence of the rumen methanogen *Methanobrevibacter ruminantium* reveals new possibilities for controlling ruminant methane emissions. PLoS ONE. 2010;5:e8926. 10.1371/journal.pone.0008926. 20126622 10.1371/journal.pone.0008926 PMC2812497

[60] Morgavi DP, Forano E, Martin C, Newbold CJ. Microbial ecosystem and methanogenesis in ruminants. Animal. 2010;4:1024–36. 10.1017/S1751731110000546. 22444607 10.1017/S1751731110000546

[61] Buan NR. Methanogens: pushing the boundaries of biology. Emerg Top Life Sci. 2018;2:629–46. 10.1042/ETLS20180031. 33525834 10.1042/ETLS20180031 PMC7289024

[62] Zheng Y, Kahnt J, Kwon IH, Mackie RI, Thauer RK. Hydrogen formation and its regulation in *Ruminococcus albus*: involvement of different hydrogenases. J Bacteriol. 2014;196:3840–52. 10.1128/jb.02070-14. 25157086 10.1128/JB.02070-14 PMC4248817

[63] Morais S, Mizrahi I. The road not taken: the rumen microbiome, functional groups, and community states. Trends Microbiol. 2019;27:538–49. 10.1016/j.tim.2018.12.011. 30679075 10.1016/j.tim.2018.12.011

[64] Myer PR, Smith TPL, Wells JE, Kuehn LA, Freetly HC. Rumen microbiome from steers differing in feed efficiency. PLoS ONE. 2015;10:e0129174. 10.1371/journal.pone.0129174. 26030887 10.1371/journal.pone.0129174 PMC4451142

[65] Jami E, White BA, Mizrahi I. Potential role of the bovine rumen microbiome in modulating milk composition and feed efficiency. PLoS ONE. 2014;9:e85423. 10.1371/journal.pone.0085423. 24465556 10.1371/journal.pone.0085423 PMC3899005

[66] Moissl-Eichinger C, Pausan M, Taffner J, Berg G, Bang C, Schmitz RA. Archaea are interactive components of complex microbiomes. Trends Microbiol. 2018;26:70–85. 10.1016/j.tim.2017.07.004. 28826642 10.1016/j.tim.2017.07.004

[67] Gonzalez-Montana JR, Escalera-Valente F, Alonso AJ, Lomillos JM, Robles R, Alonso ME. Relationship between vitamin B12 and cobalt metabolism in domestic ruminant: an update. Animals. 2020;10:1855. 10.3390/ani10101855. 33053716 10.3390/ani10101855 PMC7601760

[68] Kelly WJ, Henderson G, Pacheco DM, Li D, Reilly K, Naylor GE, et al. The complete genome sequence of *Eubacterium limosum* SA11, a metabolically versatile rumen acetogen. Stand Genomic Sci. 2016;11:26. 10.1186/s40793-016-0147-9. 26981167 10.1186/s40793-016-0147-9 PMC4791908

[69] Ungerfeld EM, Pitta DW. Biological consequences of the inhibition of rumen methanogenesis. Animal. 2024;19:101170. 10.1016/j.animal.2024.101170. 38772773 10.1016/j.animal.2024.101170

