# Supplementary material for: Rumen-derived Prevotella and Megasphaera elsdenii mitigate methane production through functional modulation of rumen microbial metabolism
Source: J Anim Sci Biotechnol. 2026 Jul 10;17:144. doi: 10.1186/s40104-026-01460-5 (PMC13352736; doi:10.1186/s40104-026-01460-5)
Supplement: Supplementary file 1 — Additional file 1: Table S1. Composition and nutritional level of diets for dairy cows. Table S2. Substrates fermentable by Prevotella and Megasphaera elsdenii. Table S3. Genomic characteristics of Prevotella and Megasphaera elsdenii. Table S4. Summary of metagenomic sequencing data from in vitro rumen fermentation samples. Table S5. The relative abundances of GH family genes encoded fibrolytic enzymes. Fig. S1. Morphological, Gram-staining, and genomic features of the isolated Prevotella and Megasphaera elsdenii strains. Fig. S2. CAZyme gene counts in Prevotella and Megasphaera elsdenii strains. Fig. S3. Functional genome annotation of Prevotella and Megasphaera elsdenii based on KEGG and COG databases. Fig. S4. Time-resolved profiles of volatile fatty acids (VFAs) and total VFA-carbon during in vitro rumen fermentation following supplementation with rumen-derived bacterial strains. Fig. S5. Eukaryotic community composition and differential analysis in rumen fermentation samples. Fig. S6. Differential abundance of Megasphaera elsdenii and functional metabolic features based on metagenomic analysis in an in vitro dairy cow fermentation system. Fig. S7. Comparative analysis of carbohydrate-active enzymes (CAZymes) in the rumen microbiome of dairy cows based on metagenomic sequencing. [file 40104_2026_1460_MOESM1_ESM.zip › Supplementary Material.docx]

| Item | Value, g/kg DM |
| --- | --- |
| Ingredient composition |  |
| Alfalfa hay | 84 |
| Oatgrass hay | 64 |
| Corn silage | 522 |
| Corn flour | 81 |
| Soybean meal | 108 |
| Whole cottonseed | 68 |
| DDGS^1^ | 48 |
| Premix^2^ | 25 |
| Total | 1000 |
| Nutritional level^3^ |  |
| CP | 15.16 |
| EE | 3.74 |
| NDF | 33.48 |
| ADF | 22.06 |
| Ash | 7.12 |
| Ca | 0.43 |
| P | 0.26 |
| NE_L_, MJ/kg^4^ | 6.32 |

Table S1 Composition and nutritional level of diets for dairy cows (DM basis)

Note: 1: DDGS: Distillers' dried grains with solubles.

2: Per kilogram of premix provides: 60 mg manganese, 80 mg zinc, 0.55 mg selenium, 75 mg iron, 35 mg copper, 1.00 mg iodine, 0.40 mg cobalt, 200,000 IU vitamin A, 30,000 IU vitamin D, and 1,000 IU vitamin E.

3: CP: crude protein, EE: ether extract, NDF: neutral detergent fiber, ADF: acid detergent fiber.

4: The NE_L_ was calculated, and the other values were measured.

Table S2 Substrates fermentable by *Prevotella* and *Megasphaera elsdenii*.

| Items^1^ | Result^2^ | | | | | |
| --- | --- | --- | --- | --- | --- | --- |
|  | RH3 | RH14 | RH27 | RH35 | RH19 |  |
| ONPG | ＋ | ＋ | ＋ | ＋ | － |  |
| Arginine | － | － | － | － | ＋ |  |
| Lysine | － | － | － | － | ＋ |  |
| Ornithine | － | － | － | ＋ | ＋ |  |
| Citric acid | － | － | － | － | － |  |
| Hydrothion | － | － | － | － | － |  |
| Urease | － | － | － | ＋ | － |  |
| Indole | － | － | － | － | － |  |
| VP | ＋ | ＋ | ＋ | ＋ | － |  |
| Gelatin | － | － | － | － | － |  |
| Gucose | ＋ | ＋ | ＋ | ＋ | ＋ |  |
| Inose | － | － | － | － | － |  |
| Sorbitol | － | － | － | － | － |  |
| Rhamnose | － | － | － | － | － |  |
| Melibiose | － | － | － | － | － |  |
| Amygdalin | － | － | － | － | － |  |
| Arabinose | ＋ | － | ＋ | － | － |  |
| Oxidase | － | － | － | － | － |  |
| Aesculin | ＋ | ＋ | ＋ | ＋ | － |  |
| Cellobiose | ＋ | ＋ | ＋ | ＋ | － |  |
| Maltose | ＋ | ＋ | ＋ | ＋ | ＋ |  |
| Mannitol | － | － | － | ＋ | ＋ |  |
| Salicin | ＋ | ＋ | ＋ | ＋ | － |  |
| Sorbitol | － | － | － | － | － |  |
| Sucrose | ＋ | ＋ | ＋ | ＋ | － |  |
| Raffinose | － | － | － | － | － |  |
| Synanthrin | ＋ | ＋ | ＋ | ＋ | － |  |
| Lactose | － | － | － | － | － |  |

Note: ^1^: ONPG; o-nitrophenyl-β-D-galactopyranoside; VP; voges–proskauer test.

^2^: “＋” Fermentable; “－” Un- fermentable. RH3 = *Prevotella* RH3; RH14 = *Prevotella* RH14; RH19 = *Megasphaera elsdenii* RH19; RH27 = *Prevotella* RH27; RH35 = *Prevotella* RH35.

Table S3 Genomic characteristics of *Prevotella* and *Megasphaera elsdenii*

| Genomic Contents^1^ | RH3 | RH14 | RH27 | RH35 | RH19 |
| --- | --- | --- | --- | --- | --- |
| Raw data, Mb | 1191.3 | 1190 | 1105.4 | 1266.9 | 1160.3 |
| Clean data, Mb | 1124.7 | 1121.7 | 1047.5 | 1196.1 | 1096.7 |
| Total scaffolds | 45 | 153 | 156 | 155 | 63 |
| Sequence length, bp | 5241519 | 4637358 | 4638824 | 4639038 | 2429558 |
| GC content, % | 37.49 | 50.48 | 50.76 | 51.12 | 53.26 |
| Gengset number | 5519 | 4514 | 4515 | 4516 | 2258 |
| Gene total length | 4490847 | 4046904 | 4047036 | 4047180 | 2121984 |
| Gene average length | 813 | 896 | 896 | 896 | 939 |
| Number of tRNAs | 67 | 73 | 77 | 79 | 55 |
| Number of 5S rRNAs | 7 | 0 | 0 | 0 | 4 |
| Number of 6S rRNAs | 0 | 1 | 1 | 1 | 1 |
| Number of 23S rRNAs | 2 | 1 | 1 | 1 | 1 |
| Total protein | 5519 | 4514 | 4515 | 4516 | 2258 |
| NR | 5329 | 4307 | 4306 | 4309 | 2151 |
| GO | 1174 | 3167 | 3168 | 3168 | 560 |
| eggNOG | 4106 | 3752 | 3752 | 3753 | 1911 |
| KEGG | 2670 | 3147 | 3147 | 3146 | 1241 |
| CAZy | 152 | 121 | 121 | 121 | 71 |

Note: RH3 = *Prevotella* RH3; RH14 = *Prevotella* RH14; RH27 = *Prevotella* RH27; RH35 = *Prevotella* RH35; RH19 = *Megasphaera elsdenii* RH19.

^1^ NR; non-redundant protein database; GO; gene ontology; eggNOG; evolutionary genealogy of genes: Non-supervised Orthologous Groups; KEGG; Kyoto Encyclopedia of Genes and Genomes; CAZy; carbohydrate-active enzymes database.

| Sample | Raw reads | Clean reads |
| --- | --- | --- |
| NC-1 | 69682530 | 69205344 |
| NC-2 | 68447452 | 67979746 |
| NC-3 | 73502074 | 73061594 |
| RH3-1 | 76261472 | 75758554 |
| RH3-2 | 70948856 | 70485814 |
| RH3-3 | 71276350 | 70798726 |
| RH14-1 | 66896626 | 66468974 |
| RH14-2 | 73563494 | 73074280 |
| RH14-3 | 69370074 | 68873394 |
| RH27-1 | 67548182 | 67164882 |
| RH27-2 | 72899544 | 72504080 |
| RH27-3 | 74393146 | 73939734 |
| RH35-1 | 72178168 | 71803092 |
| RH35-2 | 72511618 | 72122290 |
| RH35-3 | 84788338 | 84331996 |
| RH19-1 | 73064142 | 72654436 |
| RH19-2 | 72701084 | 72313042 |
| RH19-3 | 72526184 | 72150686 |
| Total | 1302559334 | 1294690664 |
| mean | 72364407 | 71927259 |
| SD | 3949254 | 3944204 |
| SEM | 930848 | 929658 |

Table S4 Summary of metagenomic sequencing data from in vitro rumen fermentation samples

Note: Raw reads represent the total number of sequencing reads obtained from each sample, while clean reads indicate the number of high-quality reads remaining after quality control and filtering. Samples are labeled according to treatment groups and biological replicates (n = 3 per group). NC = negative control; RH3, RH14, RH27, and RH35 = *Prevotella* strains; RH19 = *Megasphaera elsdenii*. Total represents the sum of all samples.

Table S5 The relative abundances of GH family genes encoded fibrolytic enzymes

| CAZymes family^1^ | Dietary treatment^2^ | | | | | | SD^3^ | *P*-value |
| --- | --- | --- | --- | --- | --- | --- | --- | --- |
|  | NC | RH3 | RH14 | RH27 | RH35 | RH19 |  |  |
| GH1 | 0.15^c^ | 0.15^c^ | 0.14^c^ | 0.16^b^ | 0.19^a^ | 0.14^c^ | 0.02 | 0.04 |
| GH2 | 6.63 | 6.74 | 6.68 | 6.68 | 6.61 | 6.72 | 0.12 | 0.44 |
| GH3 | 2.48 | 2.49 | 2.46 | 2.54 | 2.50 | 2.46 | 0.05 | 0.62 |
| GH4 | 0.07^b^ | 0.07^b^ | 0.07^b^ | 0.08^a^ | 0.08^a^ | 0.06^c^ | 0.01 | 0.02 |
| GH5 | 0.10 | 0.09 | 0.09 | 0.12 | 0.09 | 0.08 | 0.01 | 0.06 |
| GH8 | 0.24 | 0.25 | 0.25 | 0.23 | 0.25 | 0.25 | 0.01 | 0.36 |
| GH9 | 0.48 | 0.46 | 0.47 | 0.46 | 0.47 | 0.48 | 0.02 | 0.53 |
| GH10 | 0.94 | 0.98 | 0.96 | 0.89 | 0.93 | 0.98 | 0.03 | 0.06 |
| GH11 | 0.005 | 0.006 | 0.005 | 0.006 | 0.006 | 0.005 | 0.002 | 0.32 |
| GH26 | 0.74 | 0.74 | 0.73 | 0.69 | 0.71 | 0.75 | 0.03 | 0.20 |
| GH30 | 0.02 | 0.02 | 0.02 | 0.02 | 0.02 | 0.02 | 0.001 | 0.54 |
| GH31 | 0.19 | 0.18 | 0.18 | 0.19 | 0.19 | 0.19 | 0.01 | 0.89 |
| GH38 | 0.06^a^ | 0.05^b^ | 0.05b | 0.06^a^ | 0.06^a^ | 0.05^b^ | 0.005 | 0.01 |
| GH39 | 0.16^b^ | 0.15^bc^ | 0.15^bc^ | 0.20^a^ | 0.15^bc^ | 0.14^cd^ | 0.01 | 0.04 |
| GH43 | 0.05 | 0.05 | 0.05 | 0.05 | 0.05 | 0.05 | 0.003 | 0.83 |
| GH44 | 0.001 | 0.001 | 0.001 | 0.0004 | 0.001 | 0.001 | 0.0002 | 0.13 |
| GH45 | 0.001 | 0.0003 | 0.001 | 0.0003 | 0.001 | 0.001 | 0.001 | 0.19 |
| GH48 | 0.0002 | 0.0001 | 0.0003 | 0.0005 | 0.0001 | 0.0001 | 0.0002 | 0.20 |
| GH51 | 0.04 | 0.05 | 0.05 | 0.04 | 0.04 | 0.05 | 0.004 | 0.73 |
| GH52 | 0.0003 | 0.0003 | 0 | 0.0001 | 0.0001 | 0.0002 | 0.0002 | 0.47 |
| GH54 | 0.02 | 0.02 | 0.02 | 0.02 | 0.02 | 0.02 | 0.002 | 0.14 |
| GH62 | 0.0001 | 0.0003 | 0.0002 | 0.0002 | 0.0002 | 0.0004 | 0.0002 | 0.74 |
| GH67 | 0.47^b^ | 0.48^b^ | 0.48^b^ | 0.43^c^ | 0.46^b^ | 0.50^a^ | 0.011 | 0.04 |
| GH74 | 0.24 | 0.23 | 0.23 | 0.24 | 0.22 | 0.23 | 0.013 | 0.20 |
| GH92 | 1.34^ab^ | 1.25^c^ | 1.28^b^ | 1.39^a^ | 1.26^c^ | 1.21^d^ | 0.044 | 0.02 |
| GH98 | 0.05 | 0.05 | 0.05 | 0.04 | 0.05 | 0.05 | 0.005 | 0.14 |
| GH113 | 0.03^a^ | 0.03^a^ | 0.02^b^ | 0.03^a^ | 0.03^a^ | 0.02^b^ | 0.002 | 0.03 |
| GH116 | 0.01 | 0.01 | 0.01 | 0.01 | 0.01 | 0.01 | 0.003 | 0.52 |
| GH120 | 0.01 | 0.01 | 0.01 | 0.02 | 0.02 | 0.02 | 0.002 | 0.13 |
| GH124 | 0.02 | 0.02 | 0.02 | 0.02 | 0.03 | 0.02 | 0.002 | 0.64 |

Note: ^1^ GH, glycoside hydrolase. The difference among three groups was identified by the Kruskal–Wallis multiple comparisons, and the *P* < 0.05 indicated the significant difference.

^2^ NC = negative control; RH3 = *Prevotella* RH3; RH14 = *Prevotella* RH14; RH27 = *Prevotella* RH27; RH35 = *Prevotella* RH35; RH19 = *Megasphaera elsdenii* RH19. Means within a row with different superscript letters (a, b) differ significantly (*P* < 0.05).

^3^ SD, standard deviation.

Figure S1. Morphological, Gram-staining, and genomic features of the isolated *Prevotella* and *Megasphaera elsdenii* strains. (A) Colonial morphology of the isolated *Prevotella* and *Megasphaera elsdenii* strains. (B) Gram staining identification of the isolated *Prevotella* and *Megasphaera elsdenii* strains. (C) Circular genome maps of *Prevotella* and *Megasphaera elsdenii*. The outermost circle indicates genome size scale. The second and third circles represent coding sequences (CDS) on the forward and reverse strands, respectively, with colors designating functional classifications according to the Clusters of Orthologous Groups (COG) database. The fourth circle shows rRNA and tRNA locations. The fifth circle displays GC content: outward red peaks indicate regions with GC content higher than the genomic average (peak height corresponds to the degree of deviation), while inward blue peaks indicate regions with GC content lower than the genomic average. The innermost circle represents the GC skew value.



Figure S2 CAZyme gene counts in *Prevotella* and *Megasphaera elsdenii* strains.

Carbohydrate-active enzymes (CAZymes) were annotated based on the CAZy database. The y-axis represents the number of genes encoding different CAZyme families. AA, auxiliary activities; CBM, carbohydrate-binding modules; CE, carbohydrate esterases; GH, glycoside hydrolases; GT, glycosyltransferases; PL, polysaccharide lyases.


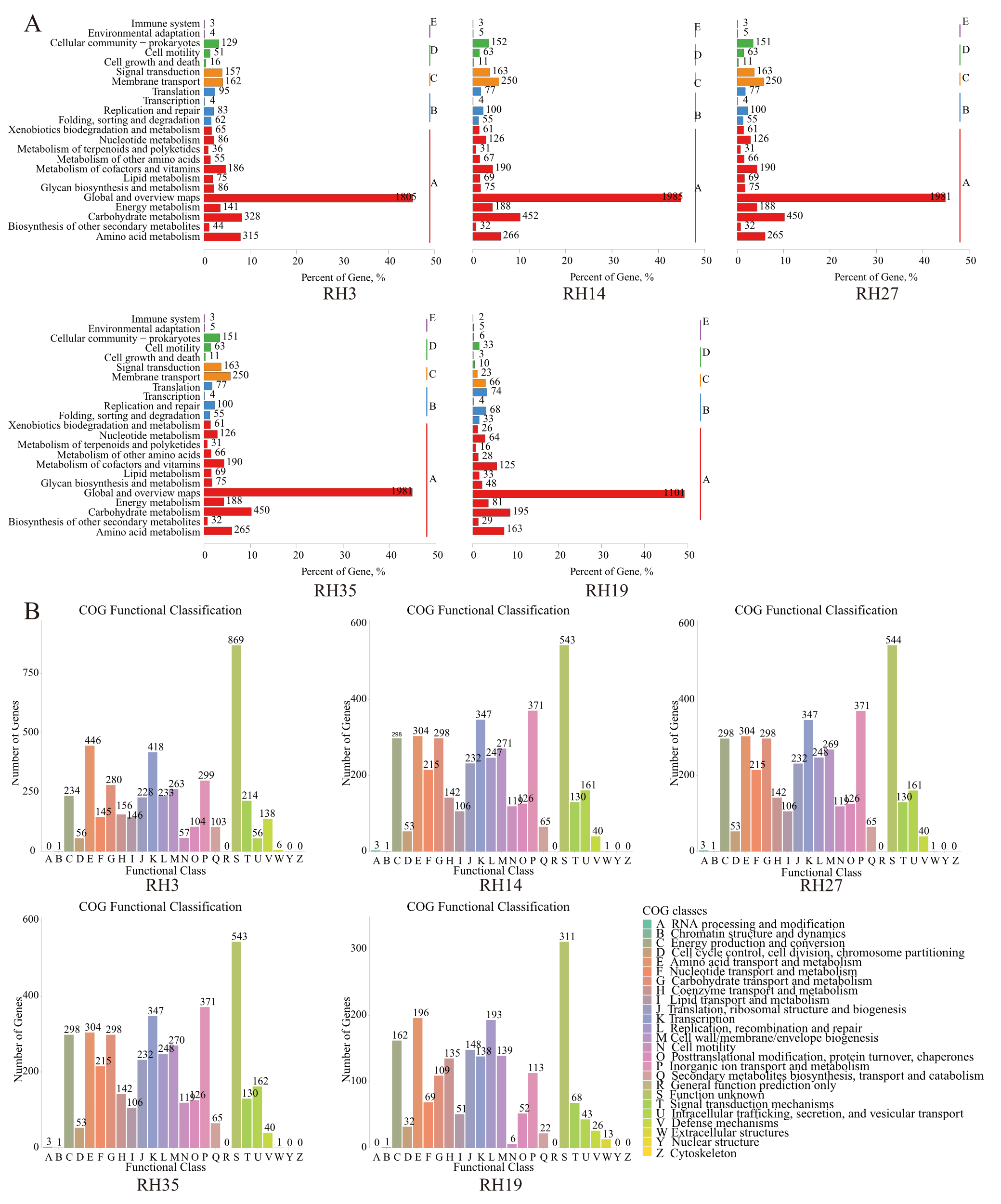
Figure S3. Functional genome annotation of *Prevotella* and *Megasphaera elsdenii* based on KEGG and COG databases. (A) Classification statistics of KEGG pathway annotations for *Prevotella* and *Megasphaera elsdenii*. The Y-axis indicates the names of KEGG metabolic pathways, while the X-axis represents both the number of genes annotated to each pathway and their proportion relative to the total number of annotated genes. (B) COG functional classification statistics for *Prevotella* and *Megasphaera elsdenii*.


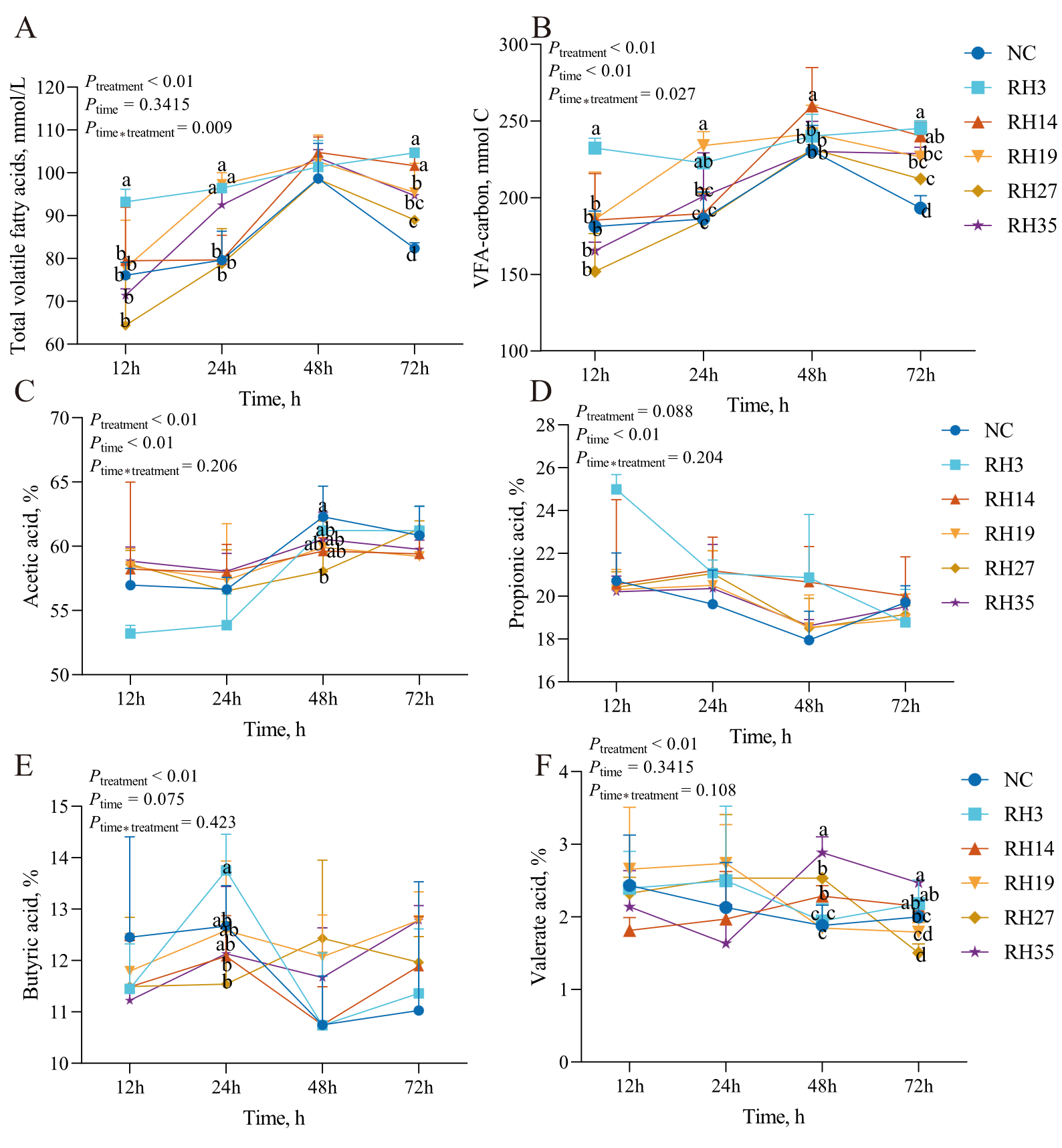


Figure S4. Time-resolved profiles of volatile fatty acids (VFAs) and total VFA-carbon during in vitro rumen fermentation following supplementation with rumen-derived bacterial strains. (A) total volatile fatty acids (TVFA, mmol/L); (B) Total VFA-carbon (mmol C/L), calculated by weighting individual VFAs according to their carbon numbers (acetate ×2, propionate ×3, butyrate ×4, and valerate ×5) and multiplying by total VFA concentration; (C) acetate (%); (D) propionate (%); (E) butyrate (%); and (F) valerate (%). Fermentation parameters were measured at 12, 24, 48, and 72 h of incubation. Treatments included a negative control (NC) and bacterial supplementation groups (RH3, RH14, RH19, RH27, and RH35). Data are presented as mean ± SEM. Different lowercase letters indicate significant differences among treatments at the same time point (*P* < 0.05). Statistical significance was assessed using two-way ANOVA with treatment and time as fixed effects, including their interaction, followed by multiple comparisons.

Figure S5. Eukaryotic community composition and differential analysis in rumen fermentation samples. (A) Relative abundance (%) of Eukaryota at the phylum, genus, and species levels across different treatment groups. NC, control group; RH3, RH14, RH19, RH27, and RH35 represent rumen fermentation treatments supplemented with different bacterial strains. Data are expressed as relative abundance (% of total sequences). The difference among six groups was identified by Kruskal–Wallis multiple comparisons, and asterisk indicated the significant difference (*P* < 0.05).


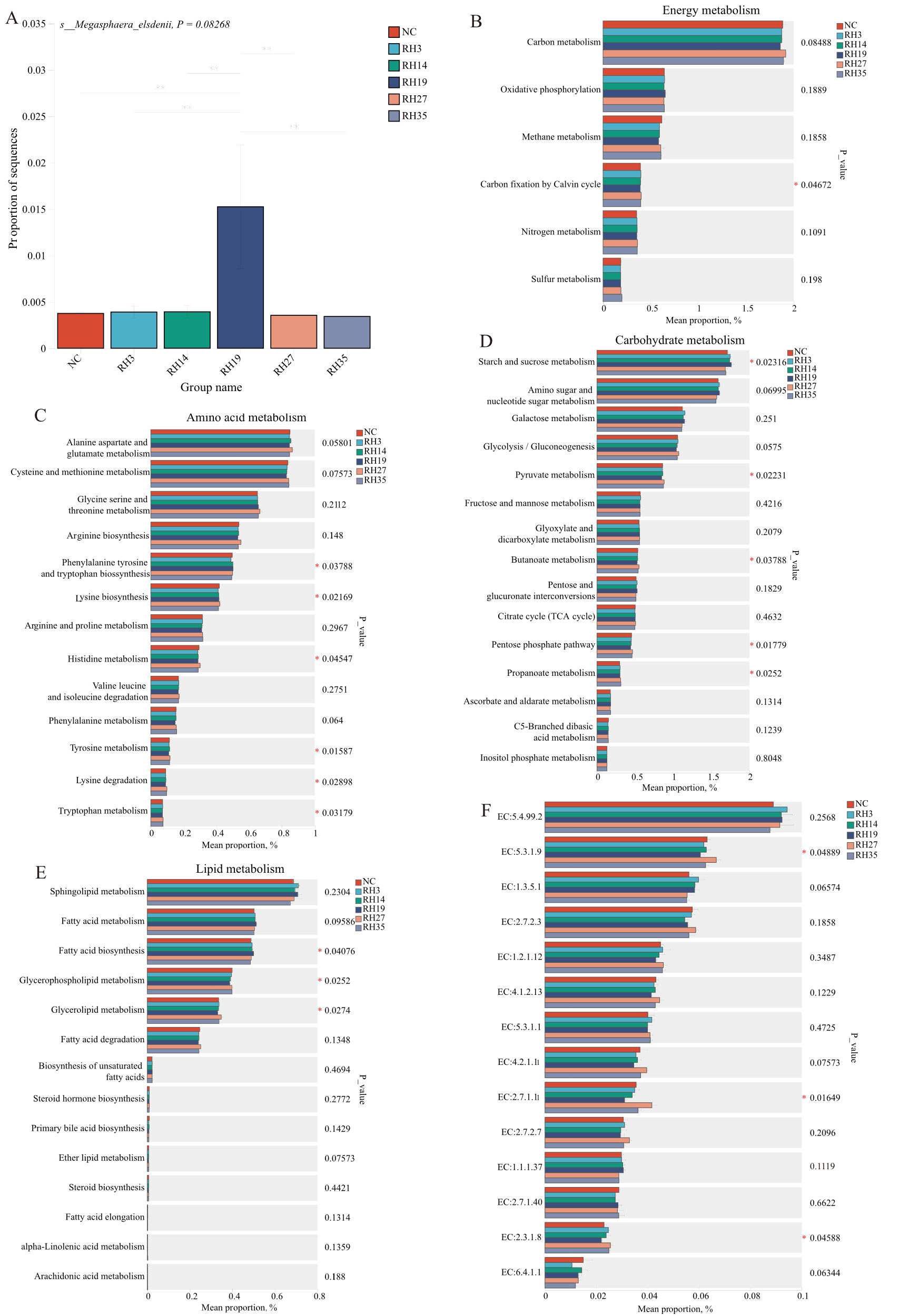


Figure S6. Differential abundance of *Megasphaera elsdenii* and functional metabolic features based on metagenomic analysis in an in vitro dairy cow fermentation system. (A) Relative abundance of *s_Megasphaera_elsdenii* across different treatment groups. (B) Comparison of the abundance of KEGG Orthology (KO) enzymes involved in acetate, propionate, and butyrate production pathways among treatments. (C) Amino acid metabolism pathways. (D) Carbohydrate metabolism pathways. (E) Lipid metabolism pathways. (F) Energy metabolism pathways. In panels (B–F), the x-axis represents the relative abundance of KEGG Orthology (KO) enzymes or pathways, and different colors indicate treatment groups (NC, RH13, RH14, RH19, RH27, RH35). Error bars represent standard deviations. Asterisks indicate statistically significant differences (**P* < 0.05, ***P* < 0.01).


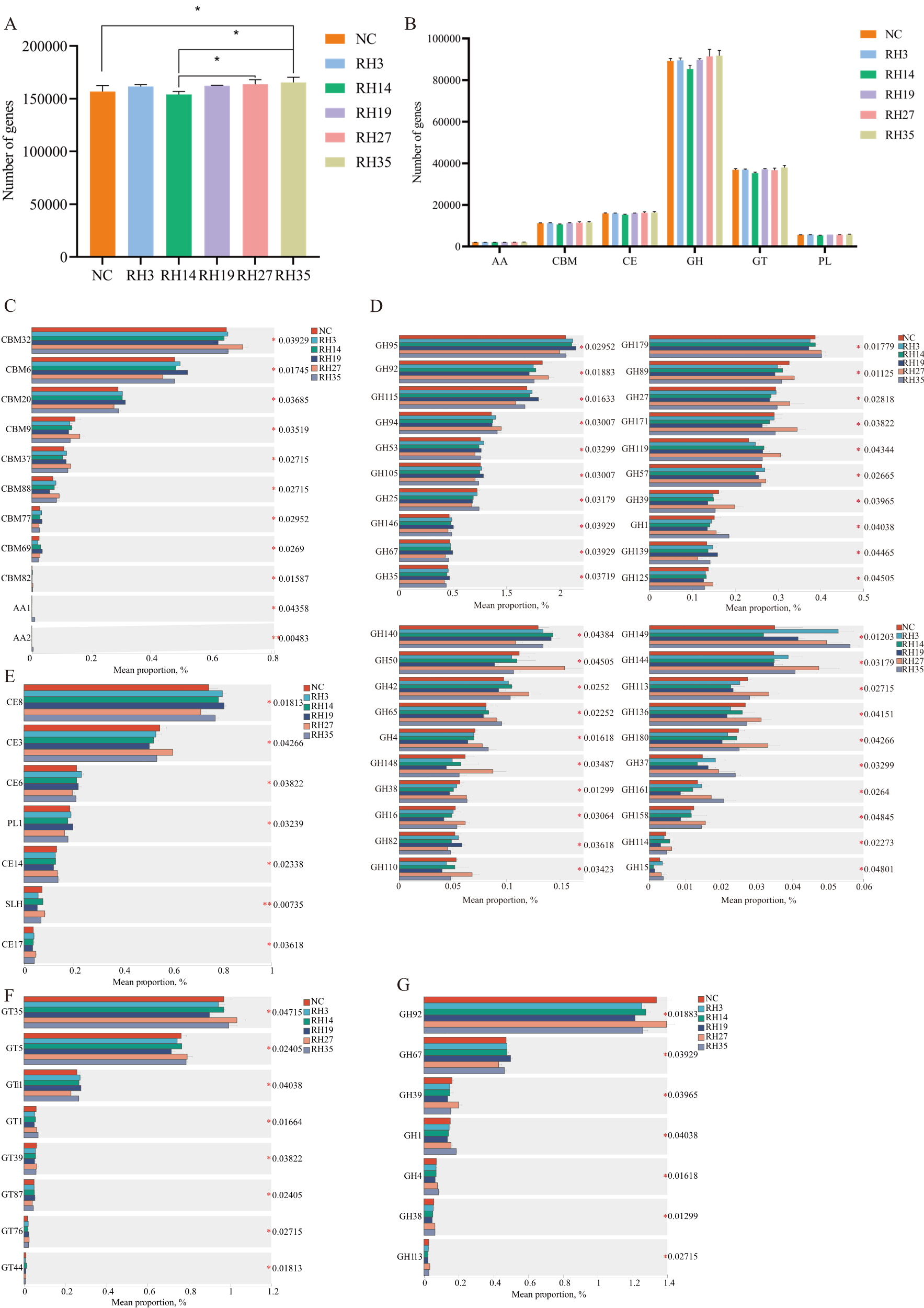


Figure S7. Comparative analysis of carbohydrate-active enzymes (CAZymes) in the rumen microbiome of dairy cows based on metagenomic sequencing. (A) Comparison of the total CAZyme gene abundance among different treatment groups. (B) Comparison of CAZyme gene family abundance among treatments. (C–F) Comparison of gene abundances for major CAZyme classes, including carbohydrate-binding modules (CBM), auxiliary activities (AA), glycoside hydrolases (GH), carbohydrate esterases (CE), polysaccharide lyases (PL), and glycosyltransferases (GT). Only families showing significant differences among groups are presented. (G) Differential abundance of CAZyme genes involved in the degradation of structural carbohydrates, including cellulose, hemicellulose, and lignin. Gene abundance was annotated based on the CAZy database. Differential abundance analysis was performed using the Kruskal–Wallis test. Error bars represent standard deviations. Asterisks indicate statistically significant differences (**P* < 0.05, ***P* < 0.01).
